# Atomistic insight into the kinetic pathways for Watson–Crick to Hoogsteen transitions in DNA

**DOI:** 10.1093/nar/gkz837

**Published:** 2019-10-30

**Authors:** Jocelyne Vreede, Alberto Pérez de Alba Ortíz, Peter G Bolhuis, David W H Swenson

**Affiliations:** Computational Chemistry, Van ’t Hoff Institute for Molecular Sciences, University of Amsterdam, Science Park 904, 1098 XH Amsterdam, The Netherlands

## Abstract

DNA predominantly contains Watson–Crick (WC) base pairs, but a non-negligible fraction of base pairs are in the Hoogsteen (HG) hydrogen bonding motif at any time. In HG, the purine is rotated ∼180° relative to the WC motif. The transitions between WC and HG may play a role in recognition and replication, but are difficult to investigate experimentally because they occur quickly, but only rarely. To gain insight into the mechanisms for this process, we performed transition path sampling simulations on a model nucleotide sequence in which an AT pair changes from WC to HG. This transition can occur in two ways, both starting with loss of hydrogen bonds in the base pair, followed by rotation around the glycosidic bond. In one route the adenine base converts from WC to HG geometry while remaining entirely within the double helix. The other route involves the adenine leaving the confines of the double helix and interacting with water. Our results indicate that this outside route is more probable. We used transition interface sampling to compute rate constants and relative free energies for the transitions between WC and HG. Our results agree with experiments, and provide highly detailed insights into the mechanisms of this important process.

## INTRODUCTION

**Figure 1.**
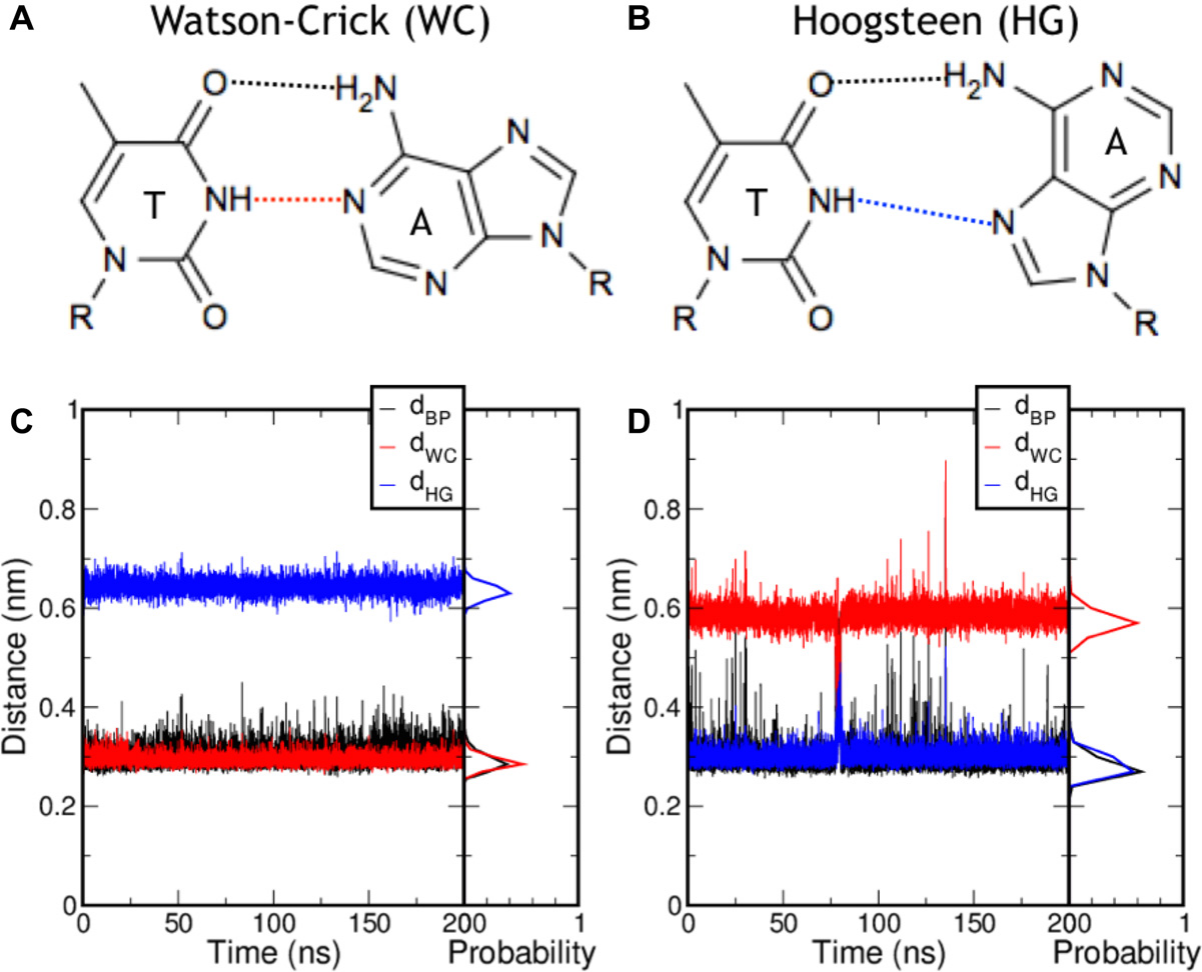
Hydrogen bond patterns for an adenine–thymine (AT) base pair. (A) and (B) show chemical drawings in (**A**) Watson–Crick (WC) and (**B**) Hoogsteen (HG) configuration. (C) and (D) show time traces for the hydrogen bond distances of a 200 ns MD simulation in (**C**) WC and (**D**) HG, with probability histograms showing the accumulated data for (**C**) 9 and (**D**) 5 individual 200 ns MD runs. The hydrogen bond distances *d*_BP_, *d*_WC_ and *d*_HG_ are colored black, red and blue, respectively.

Six years after Watson and Crick published their model for the structure of DNA (1), Karst Hoogsteen suggested an alternative way for nucleotide bases to form hydrogen bonded pairs (2). This alternative geometry has the purine flipped ‘upside-down’, such that the 5-ring of the purine forms a hydrogen bond to the pyrimidine, rather than the 6-ring (Figure 1 A and B). In DNA, going from the Watson–Crick (WC) to Hoogsteen (HG) geometry, the flip of the purine (from *anti* in WC to *syn* in HG) requires a ∼ 180° rotation of the base along the bond connecting the base to the sugar, known as the glycosidic bond. For more detail on the structural aspects see the Supporting Information, including Supplementary Figure S1. Raman spectroscopy of a Hoogsteen AT base pair in a crystalline environment revealed altered vibrations for atoms participating in the hydrogen bonding interactions between the base pairs (3). Advanced dispersion NMR experiments uncovered the transient presence of HG geometries in naked duplex DNA (4), suggesting that a non-negligible amount of DNA exists in the HG configuration. Recently, the sequence d(ATTAAT)_2_ was shown to crystallize in a double helix with all the bases in HG geometry, indicating that any AT-rich sequence may contain HG base pairs (5). A survey of known protein–DNA crystal structures revealed that HG base pairing occurs in several protein–DNA complexes (6), including the human DNA polymerase hPol-iota (7), involved in replication. This particular polymerase replicates DNA via HG base pairing (7). Biochemical assays show that hPol-iota is severely inhibited by modified nucleotides that cannot adopt the HG conformation (8), thus elevating HG base pairing to a position so far only reserved for WC base pairs: they provide a basis for duplicating DNA (9). In addition, Hoogsteen base pairing plays a role in cell division and differentiation, as indicated by crystal structures of the anti-tumor protein p53 (10) and the cell differentiation regulating MAT α2 homeodomain (11), both in complex with DNA containing HG base pairs. These structures provide evidence for the biological relevance of Hoogsteen base pairs, yet little is known on the mechanisms of the conversion between WC and HG base pairs.

In naked DNA, one out of every ∼250 AT base pairs occurs in the HG configuration at any given time, with a residence time of several hundreds of μs (4). This transient nature of the HG configuration hampers thorough experimental investigation of the mechanism of WC to HG conversion. Molecular simulation can complement experiments by providing atomistic insights at high spatial and temporal resolution. Previous work involved quantum chemical calculations which predicted that the stability of AT base pairs in HG configuration would be comparable to that of WC pairing in DNA duplexes (12). Recent discrete path sampling computations suggest that the transition of a DNA duplex containing only WC base pairs to a duplex exclusively containing HG base pairs involves several intermediates related to the conversion of single base pairs (13). However, the mechanism of single WC to HG conversions was not considered in that study (13).

Currently, a brute-force all-atom molecular dynamics (MD) investigation of the mechanism and kinetic aspects of the WC to HG transition is impossible, as the long timescales, in the order of several milliseconds, are prohibitive for such studies. Such long timescales are caused by high free energy barriers separating the stable states. One way of overcoming these barriers is by employing biasing potentials that drive the system towards the barrier region along a predefined reaction coordinate. Two earlier computational studies using biasing methods suggested that the WC to HG transition in a single base pair can occur in various ways. Using conjugate peak refinement (14) several mechanisms for the WC to HG transition were identified, yet this study did not include explicit water molecules, thus providing little evidence as to what the most likely mechanism would be (4). The existence of multiple possibilities for the WC to HG conversion was also suggested by umbrella sampling simulations (15), yet required the backbone of the DNA to be restrained to prevent distortion by the biasing potentials. While methods employing additional potentials are well suited for computing free energy barriers and other thermodynamic properties, they often fail to yield mechanistic insight at ambient conditions, as a poor choice of reaction coordinate may lead to a wrong reaction mechanism, bad sampling and a poor estimation of the rate constants.

The transition path sampling (TPS) (16) algorithm is another way to solve the timescale problem which avoids these drawbacks. TPS collects an ensemble of short reactive trajectories connecting a predefined initial and final state, without prior knowledge of the transition state region. The definitions of the initial and final states can be very simple. For instance, the WC and HG states only require order parameters related to the hydrogen bond patterns between the bases in the base pair undergoing the transition. By defining interfaces along an order parameter, which tracks the progress of the transition, transition interface sampling (TIS) enables the calculation of rates and relative free energies (17). More importantly, the speed-up gained by using TPS and related techniques is tremendous. Assuming a transition rate in the order of 10 s^−1^, observing a single transition would require on average 100 ms of molecular dynamics. For a reasonably accurate estimation of the rate constant many transitions are required, thus scaling up the required simulation time to seconds. In contrast, when using TPS, the barrier region is sampled using MD trajectories of only tens of nanoseconds, thus providing a speed-up in the order of several million to a billion.

TPS enables analysis of the mechanism, the transition state ensemble and the reaction coordinate in terms of order parameters (18). It has been used to study the base ‘flipping’ transition in a small DNA oligomer (19), and here we apply TPS to study the transitions between the WC and HG base-pairing motifs. Our results reveal that WC to HG conversion can proceed along several mechanistic routes with varying degrees of exposure of the purine to solvent. The most prevalent route involves the adenine base flipping out of the double helix into the solvent, followed by rotation along the glycosidic bond and re-entry into the double helix. This finding contrasts with earlier computational studies, which found the route with the base remaining inside the helical confines to be more likely. These studies used either an implicit water model (4,13) or restrained the movement of the DNA backbone (15). Using TIS (17), we computed rates for the WC to HG transition, in agreement with experimental data (4). Our results provide a basis to expand the investigation of WC to HG transitions in different nucleotide sequences (20,21), including modified nucleotides (22,23), and in the presence of various other factors, such as DNA binding proteins, ions and compounds affecting DNA, such as formaldehyde (24) and triostin A (25). Futhermore, our models may aid in the interpretation of experimental data by identifying intermediate states in spectroscopic studies (26).

## MATERIALS AND METHODS

From (4), we selected nucleotide sequence 5′-CGATTTTTTGGC-3′ in which the ninth pair (T9-A4’) undergoes the WC to HG transition. Preparation of the system and all MD simulations were performed with GROMACS, version 4.5.3 (27), employing the AMBER03 force field (28) in combination with the TIP3P water model (29). Transition path sampling (TPS) (16,18) carries out an unbiased sampling of MD trajectories that connect predefined initial and final states, in the current case the WC and HG configurations as defined by different hydrogen bonding patterns. TPS is a random walk through trajectory space, where new trajectories are generated from old trajectories by a shooting algorithm, and accepted with a Metropolis rule (16,18). Here, we use the one-way, flexible path length TPS algorithm as previously implemented and applied to protein systems (30). Rate constants can be computed with transition interface sampling (TIS) (17). Like TPS, this method is a Monte Carlo procedure in path space. TIS involves ‘interfaces’ or hypersurfaces for a fixed value of some order parameter λ, where the order parameter should be a reasonable approximation of the progress of the transition. As λ we used ${\rm arctan2}(d_{\rm {WC}},d_{\rm {HG}})$, with *d*_WC_ and *d*_HG_ specific hydrogen bond distances for WC and HG respectively, to sample both the WC to HG and the HG to WC transitions. Further details on these methods can be found in the Supporting Information, including Supplementary Figure S2. Data and custom scripts are available upon request.

## RESULTS AND DISCUSSION

### Preferred mechanism for the Watson–Crick to Hoogsteen transition

The transition from Watson–Crick to Hoogsteen base pairing can occur for the pair at T9 in the sequence 5′-CGATTTTTTGGC-3′ as observed in relaxation dispersion NMR experiments (4). We performed nine independent 200 ns MD simulations on this nucleotide sequence with all base pairs in Watson–Crick (WC) configuration. Figure 1C shows the time traces of the hydrogen bond distances *d* of one of these simulations, with *d*_BP_ the hydrogen bond that is formed in both WC and HG, *d*_WC_ the hydrogen bond that forms when the base pair is in WC configuration and *d*_HG_ the hydrogen bond that is formed when the base pair is in Hoogsteen (HG) configuration; see Figure 1 A and B for a molecular drawing. Figure 1 C also shows the probability histograms for the *d*_WC_ and *d*_HG_ distances of the 1.8 μs aggregate simulation time. A similar observation can be made for the system with the T9-A4’ base pair in the HG configuration, see Figure 1D for hydrogen bond distance time traces of a 200 ns MD run and probability histograms of five independent 200 ns MD simulations. Though the Hoogsteen state exhibits larger fluctuations in the hydrogen bond distances, no transitions occur within the 1 μs aggregate simulation time. As no transitions between WC and HG are observed in the μs aggregate simulation time, such transitions can be considered rare and occur on timescales several orders of magnitude separated from the molecular timescale.

**Figure 2.**
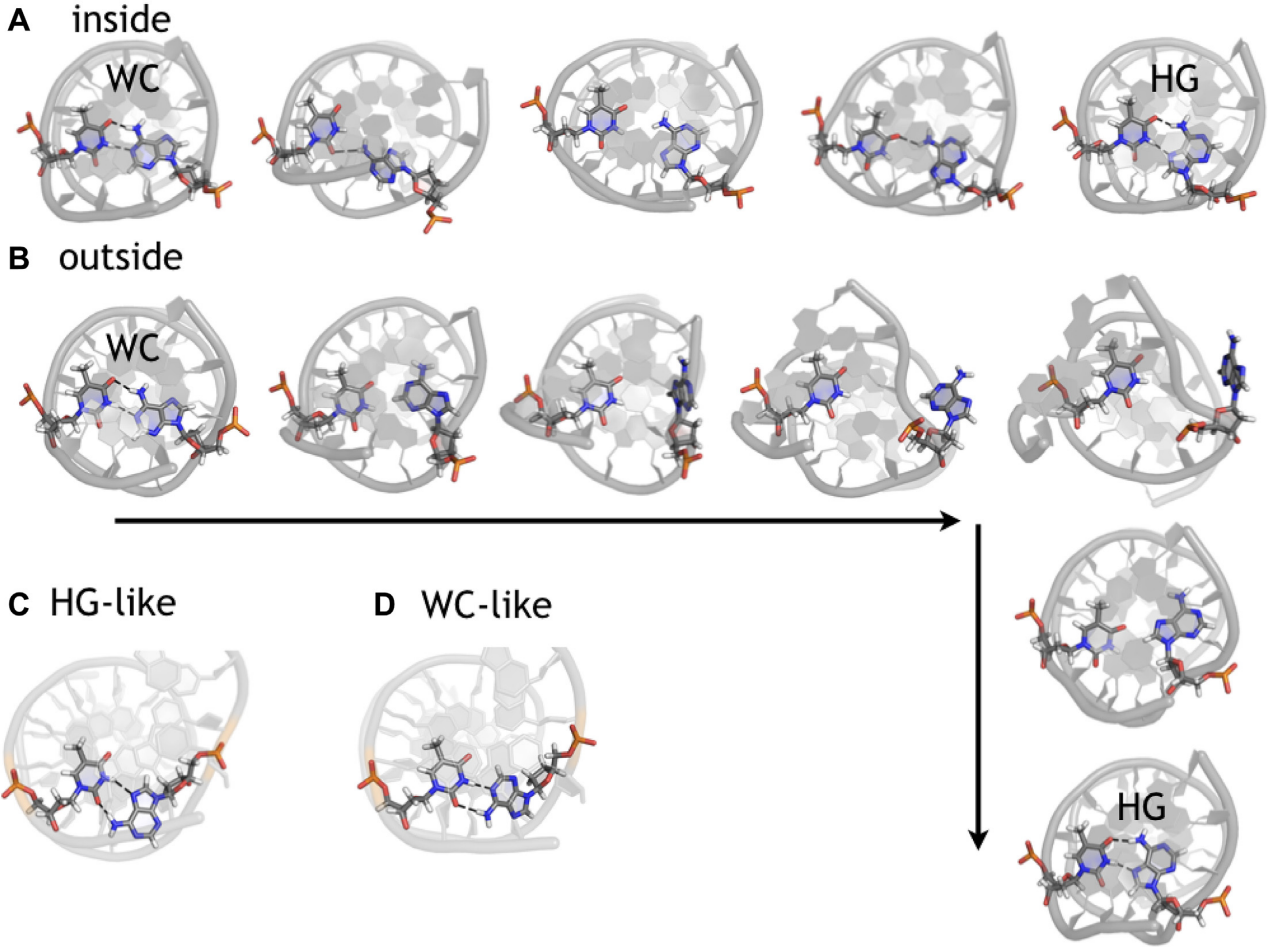
Snapshots of transition paths. Snapshot series showing (**A**) the *inside* and (**B**) the *outside* routes. Snapshots of the additional states (**C**) WC-like and (**D**) HG-like. The DNA is rendered as a grey cartoon model, with the A-T base pair undergoing the transition in sticks with carbon in gray, nitrogen in blue, oxygen in red, phosphorus in orange and hydrogen in white. The black dashed lines indicate hydrogen bonds.

The transition path sampling (TPS) approach avoids long waiting times in stable states by focusing on the actual transition between those states. Starting from an initial trajectory that samples a WC to HG transition, TPS collects reactive trajectories connecting WC and HG by monitoring MD simulations. These are started from randomly chosen snapshots along a reactive trajectory called shooting points. If the MD simulation reaches a stable state, the trial trajectory can be accepted if it connects WC to HG. By first forcing a transition using metadynamics (31), followed by equilibration of this transition to unbiased dynamics (see the Supporting Information), we were able to investigate two types of transitions occurring for the WC to HG conversion: *inside* and *outside*. In *inside* transitions, the adenine base converts from WC to HG while remaining inside the double helix, while in *outside* transitions the base flips outward into the solvent before entering the HG state. Figure 2A and B shows representative snapshots of an *inside* and an *outside* path respectively. Using both transition types as initial paths, we initiated several independent path sampling runs, labeled *inTPS* when started from the *inside* transition and *outTPS* when started from the *outside* transition, resulting in 1880 accepted paths for the *inTPS* simulations and 1716 accepted paths for the *outTPS* simulations. The acceptance ratio for both sets was around 30%. For both sets, the most common path length is ∼1.8 ns. For details on the path sampling statistics, see Supporting Information, Supplementary Table S1 and Supplementary Figure S4. A path tree, such as shown in the Supporting Information, Supplementary Figure S5, shows that distribution of shooting points in either WC (green) or HG (red) direction is balanced, indicating good sampling, and that paths are sufficiently decorrelated. The total number of decorrelated paths is ∼125.

**Figure 3.**
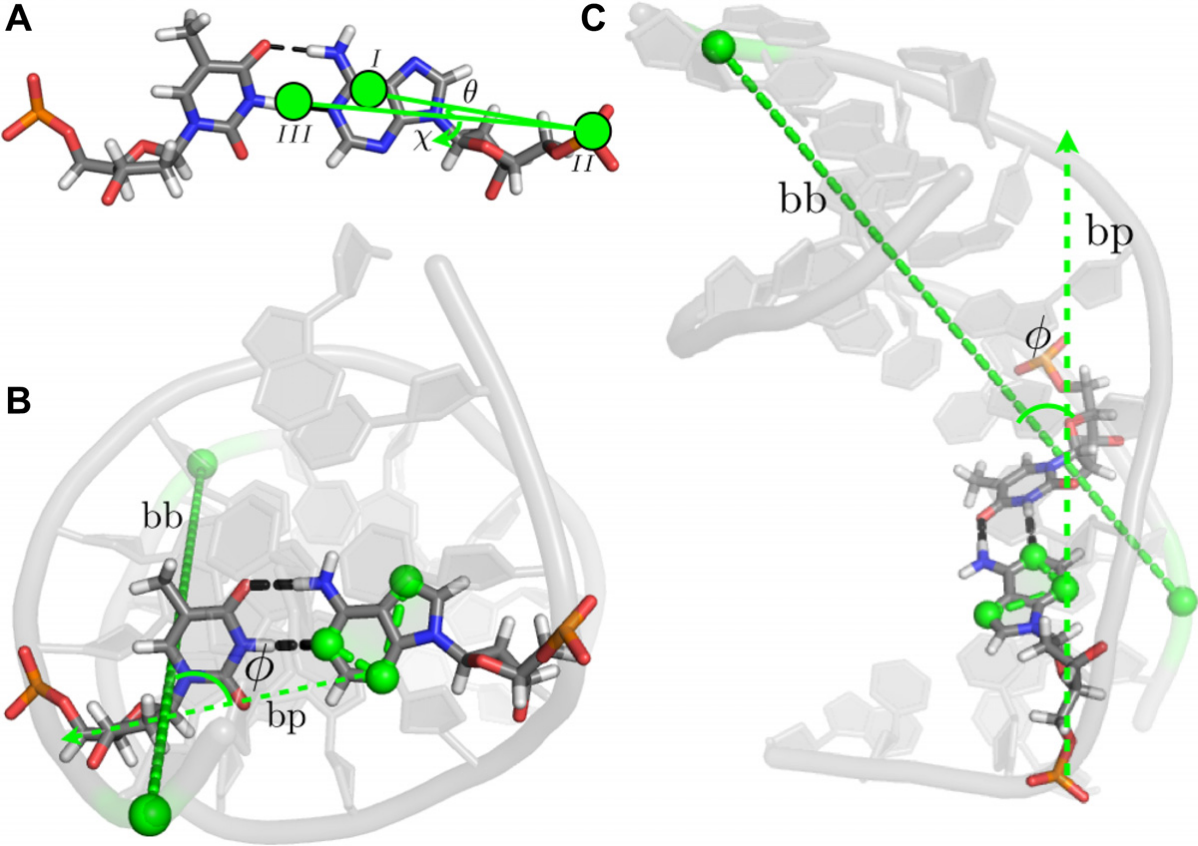
Schematic illustration of relevant order parameters. (**A**) The glycosidic angle χ and the base opening angle θ. (B, C) The base rolling angle ϕ from the top (**B**) and side (**C**) of the DNA. χ is the dihedral angle between atoms O4’, C1’, N9, C4 in the rolling base 4DA’. θ is the angle between I. the center of mass of the rolling base, II. the center of mass of the phosphate groups in nucleotides 4DA’ and 5DA’, directly neighboring the nucleotide containing the rolling base and III. the center of mass of the base pairs 8DT-5DA’ and 10DG-3DC’, directly neighboring the base pair containing the rolling base. This definition is taken from Ref. (32). ϕ is the angle between the vectors *bp* and *bb*, which is a proxy for the long axis of the DNA. Vector *bb* connects the phosphorus atoms in residues DG11 and DC11’. Vector *bp* is the vector resulting from the cross product of the vectors connecting atoms N3 and N1 and N3 and N7 in the rolling base.

For all trajectories several geometric parameters were calculated, including the hydrogen bond distances between the bases *d*_WC_ and *d*_HG_, the number of water oxygen atoms within 0.6 nm of atom N6 of residue 4DA’ *N*_water_, the base opening angle θ and the base rolling angle ϕ, see Figure 3. The latter turned out to distinguish WC from HG better than the glycosidic angle χ, as in ϕ only the orientation of the base is considered, see the Supporting Information, Supplementary Figure S3. The hydrogen bond distances *d*_WC_ and *d*_HG_ can be plotted as a single coordinate in the form of $\lambda = {\rm arctan2}(d_{\rm WC},d_{\rm HG})$. The Supporting Information contains a detailed description of all the order parameters. The distance *d*_WC_ is 0.3 nm in the WC state, and 0.6 nm in HG, and vice versa for *d*_HG_, which corresponds to λ = 0.46 and 1.11 respectively.

**Figure 4.**
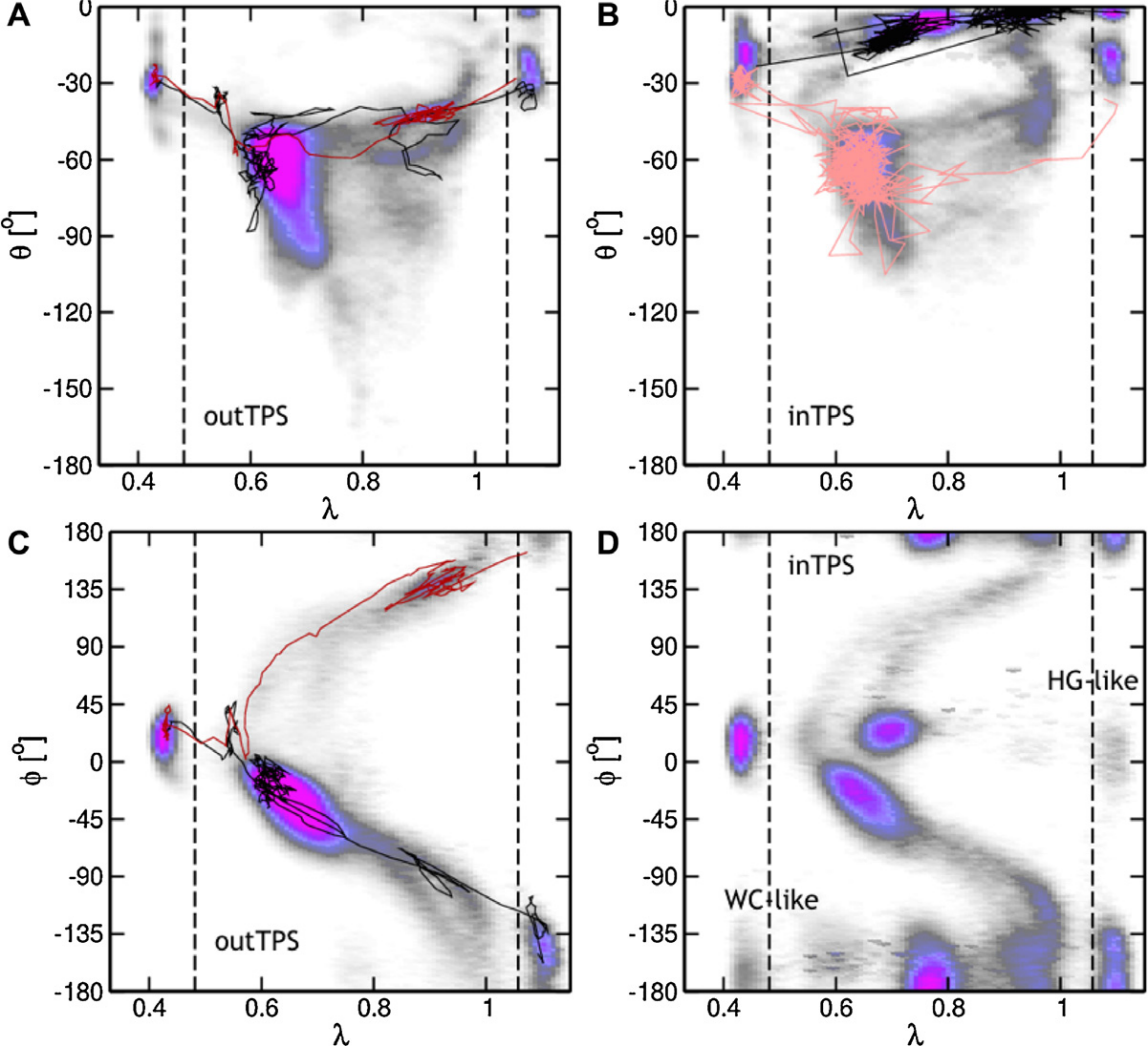
Path density plots in the λ, θ plane (**A**, **B**) and the λ, ϕ plane (**C**, **D**) for the (A, C) *outTPS* and (B, D) the *inTPS* simulations. The color of the path density ranges from white (no paths) to magenta (highest path density). The black, red and pink lines indicate representative paths as a guide to the eye. The dashed lines indicate the boundaries for the stable states as projected onto λ.

To obtain insights into the mechanistic aspects of the WC to HG conversion, the path ensembles are projected as a path density onto two-dimensional planes defined by order parameters, such as described in the previous paragraph. See the Supporting Information for further details on how the path density is obtained. For both sets of simulations we plotted the path density projected onto λ, the base rolling angle ϕ and the base opening angle θ in Figure 4. θ is close to 0° in both WC and HG states, indicating the adenine base is within the double helix, and pairing with thymine T9 in the opposite strand. The base rolling angle ϕ has a value of around 10° in the WC state and ranges between 135° and −135° including the periodic boundary at ±180°. The path densities in the λ,θ plane show striking differences for the *inTPS* and the *outTPS* simulations, see Figure 4 A and B. The path density obtained for the *outTPS* simulations shows that θ lowers to −30° before an increase occurs at λ = 0.5, indicating departure from the WC state. Similarly, upon leaving the HG state as indicated by λ < 1.0, θ has reached −20°. When λ is in between [0.5,0.9], the base opening angle is never close to zero, but −30° or lower, with the majority of paths reaching −60° to −90°. These values for the base opening angle represent paths with the adenine leaving the confines of the double helix. For the *inTPS* simulations, a similar pattern emerges, with the addition of density occurring at θ > −30°. This density indicates paths with the adenine staying within the double helix. Comparing the two density plots shows that the two TPS simulations have not converged to the same profile. Both the *outTPS* and the *inTPS* simulations show density indicating *outside* paths, however, the *outTPS* does not sample any *inside* paths. We conclude that the WC to HG conversion can occur in two ways, either *inside* or *outside*, and as a first qualitative assessment *outside* seems to be more likely.

The path density plots in the λ,ϕ plane indicate that the adenine can rotate in two directions, see Figure 4C and D. An increase or a decrease in ϕ indicates clockwise or counter-clockwise rotation looking along the axis of the glycosidic bond from the base to the deoxyribose group. The lines in Figure 4C indicate representative paths showing both types of rotation. An intermediate conformation at around λ = 0.7, ϕ = [−60°, 0°] occurs, indicating the counter-clockwise route (black line in Figure 4C) seems to be favored over the clockwise rotation, or that a partially rotated intermediate state is visited. The *outTPS* simulations sample only *outside* paths. The *inTPS* simulations must sample *inside* paths, but the path densities show striking similarities to the *outTPS* density. However, additional density occurs at λ = 0.7, ϕ = [0°, +45°] and λ = 0.75, ϕ = [−180°, −135°] and [135°, 180°] representing rotation within the double helix. No configurations in between these ranges were observed, indicating that this rotation occurs faster than the time between consecutive frames in the trajectories, which is 5 ps. Therefore, we could not identify *inside* transitions as clockwise or counter clockwise. However, the path length distributions of both sets of TPS simulations peak at 1.5 ns, which is much longer than the time required for rotation of the base, see Supplementary Figure S4 in the Supporting Information. The rotation of the base seems to be the fastest step in the *inside* route, preceded by hydrogen bond breakage and followed by hydrogen bond reformation, both of which are slower. Finally, additional density seems to occur in Figure 4D at λ < 0.48 and at λ > 1.1, indicating conformations that are either WC-like, or HG-like, respectively. Snapshots of these conformations are shown in Figure 2C and D for HG-like and WC-like respectively. The HG-like conformation shows the adenine in WC orientation, but forming a different hydrogen bonding pattern to the thymine, involving oxygen atom O2 rather than O4 of the thymine. Similar for the WC-like conformation, the adenine is in HG conformation, but forming a hydrogen bond to the oxygen atom O2.

**Figure 5.**
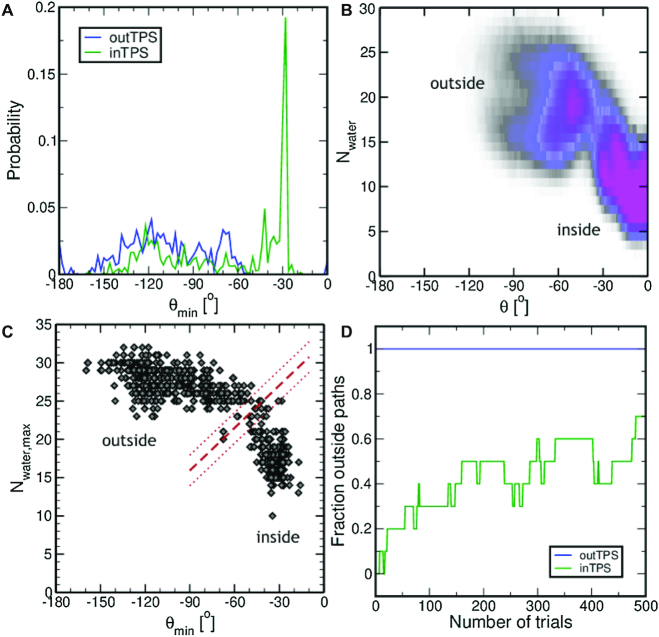
Analysis of outside and inside paths. (**A**) Path-weighted probability histogram of the minimum base opening angle θ_min_ for the *inTPS* and *outTPS* simulations. (**B**) Path density plot in the θ, *N*_water_ plane for the *inTPS* simulations. The color of the path density ranges from white (no paths) to magenta (highest path density). (**C**) Scatter plot of θ_min_ and *N*_water, max_ for each accepted path in the *inTPS* simulations. The dashed red line indicates the dividing line between *inside* and *outside* paths. The dotted red lines indicate the region in which paths are considered to be neither *inside* nor *outside*. (**D**) Fraction of *outside* paths per trial.

To distinguish between *inside* and *outside* paths we plotted histograms of θ_min_, the minimum value of θ, in each path for the *inTPS* and the *outTPS* simulations, see Figure 5A. The paths in the *outTPS* simulations do not sample θ_min_ > −50°, while the paths in the *inTPS* simulations go up to θ_min_ = −22°. In the *outside* route, the adenine residue undergoing the transition becomes exposed to solvent. An alternative way of distinguishing *inside* and *outside* paths could be provided by counting the number of water molecules around the rolling base. The Supporting Information, in particular Supplementary Figures S6, S7 and related text, includes a discussion about tuning the calculation of *N*_water_ such as to give a sufficiently clear separation of *inside* and *outside* channels. Figure 5B shows the path density of the *inTPS* simulations projected onto θ and the number of water oxygen atoms within a radius of 0.6 nm of atom N6 of the rolling adenine, *N*_water_. The path density shows two channels, one centered at *N*_water_ = [10] and θ > −34°, and one centered at *N*_water_ = 20 and θ = −50°. These channels are not fully separated. We then plotted θ_min_ and the maximum value for *N*_water, max_ in Figure 5 C. This scatter plot suggests that there are two sets of paths, which overlap. These sets can be separated by a linear function *N*_water, max_ = 0.185θ_min_ + 32, as indicated by the red line in Figure 5C. Paths below this line are *inside* paths, and paths that are above the dividing line are *outside* paths. This dividing line was fitted to two points in the region of the lowest density in between *inside* and *outside*. We also defined a region in which paths are classified as neither *inside* nor *outside* with a margin of *N*_water,max_ = 2 above and below the dividing line, see Figure 5C. The Supporting Information contains a discussion on why a margin of *N*_water,max_ = 2 is sufficient. With this definition for *inside* and *outside*, we can now track whether the TPS simulations switch from sampling *inside* paths to *outside* paths and vice versa. Note that if a path is classified as neither *inside* nor *outside* it is assigned the label of the previously accepted path. The path length distribution, shown in Supplementary Figure S4 in the Supporting Information, shows that *inside* paths are slightly shorter than *outside* paths. Both the *inTPS* and *outTPS* simulations consisted of 10 independent runs. Figure 5D shows the fraction of *outside* paths as a function of the number of trials. The *outTPS* simulations starts with all runs containing *outside* paths and never switches to sampling *inside* paths. For the *inTPS* simulations the fraction of *outside* paths is zero at the beginning, and increases to 0.7 after 500 trials, indicating that most runs switch to sampling *outside* paths. This observation strongly suggests that the *outside* path is the predominant mechanism for the WC to HG transition.

We can quantify this statement by a rough statistical analysis. We assume that the switching process is a Markov process with a probability $p({ \rm {inside} \rightarrow \rm {outside}| TP_{\rm {in}}})$ to undergo the switching from *inside* to *outside*, and the probability $p({ \rm {outside} \rightarrow \rm {inside}| TP_{\rm {out}}})$ for the reverse process. When starting in the *inside* channel, this process relaxes to the equilibrium distribution which would be dominated by the *outside* paths. We can estimate the relaxation time (in terms of trials) by fitting the green curve in Figure 5D to an exponential function, *p*_outside_ = 1 − e^*t*/τ^, which roughly gives a relaxation time of τ = 350 trials moves. Thus we can conclude that $p({ \rm {inside} \rightarrow \rm {outside}| TP_{\rm {in}}}) \approx 1/350$.

To show that the *outside* channel is more likely than the *inside* one, we need to show that $p({ \rm {inside} \rightarrow \rm {outside}| TP_{\rm {in}}}) >p({ \rm {outside} \rightarrow \rm {inside}| TP_{\rm {out}}})$. Given the evidence that we have observed seven out of ten switches from *inside* to *outside*, while at the same time the number of observed *outside* to *inside* switches remains zero, a simple Bayesian analysis yields the probability that this is true equals ∼0.99948, see the Supporting Information. Thus, the odds in favor of the hypothesis that the *outside* channel is more stable than the *inside* channel, are ∼2000 against 1.

### Rate constants for the Watson–Crick to Hoogsteen transition

Transition Interface Sampling (TIS) is a path sampling technique to calculate reaction rates. TIS requires the definition of interfaces along an order parameter that is a reasonable estimate of the reaction coordinate (17). To calculate the rates of the Watson–Crick to Hoogsteen transition using TIS, the same stable states definitions as used in the TPS simulations can be used. However, neither the glycosidic angle χ nor the base rolling angle ϕ can be used as a order parameter to track the transition, even though both angles have distinctly different values in the WC and the HG states. In particular for the outside transition, χ and ϕ can take any value as the adenine group can rotate freely once it has left the interior of the double helix. Instead we used the $\lambda = {\rm arctan2}(d_{\rm {WC}},d_{\rm {HG}})$ parameter, as this provides distinction between the stable states and a sufficient description of the progression of the transition. To determine whether λ is a reasonable order parameter to track the progression of the WC to HG conversion, and vice versa, we plotted path density profiles for each interface in the λ, θ and the λ, ϕ planes, in the Supporting Information, Supplementary Figures S9– S12. Interfaces close to the final state show θ far from zero, indicating that those interfaces sample *outside* transitions. Interfaces close to the initial states do not sample such large ranges for θ, indicating that the distinction between *inside* and *outside* becomes relevant only after a certain progression of the transition. The path density profiles suggest that the choice for going via the *inside* or the *outside* channel lies at 0.65 <λ < 0.75.

**Figure 6.**
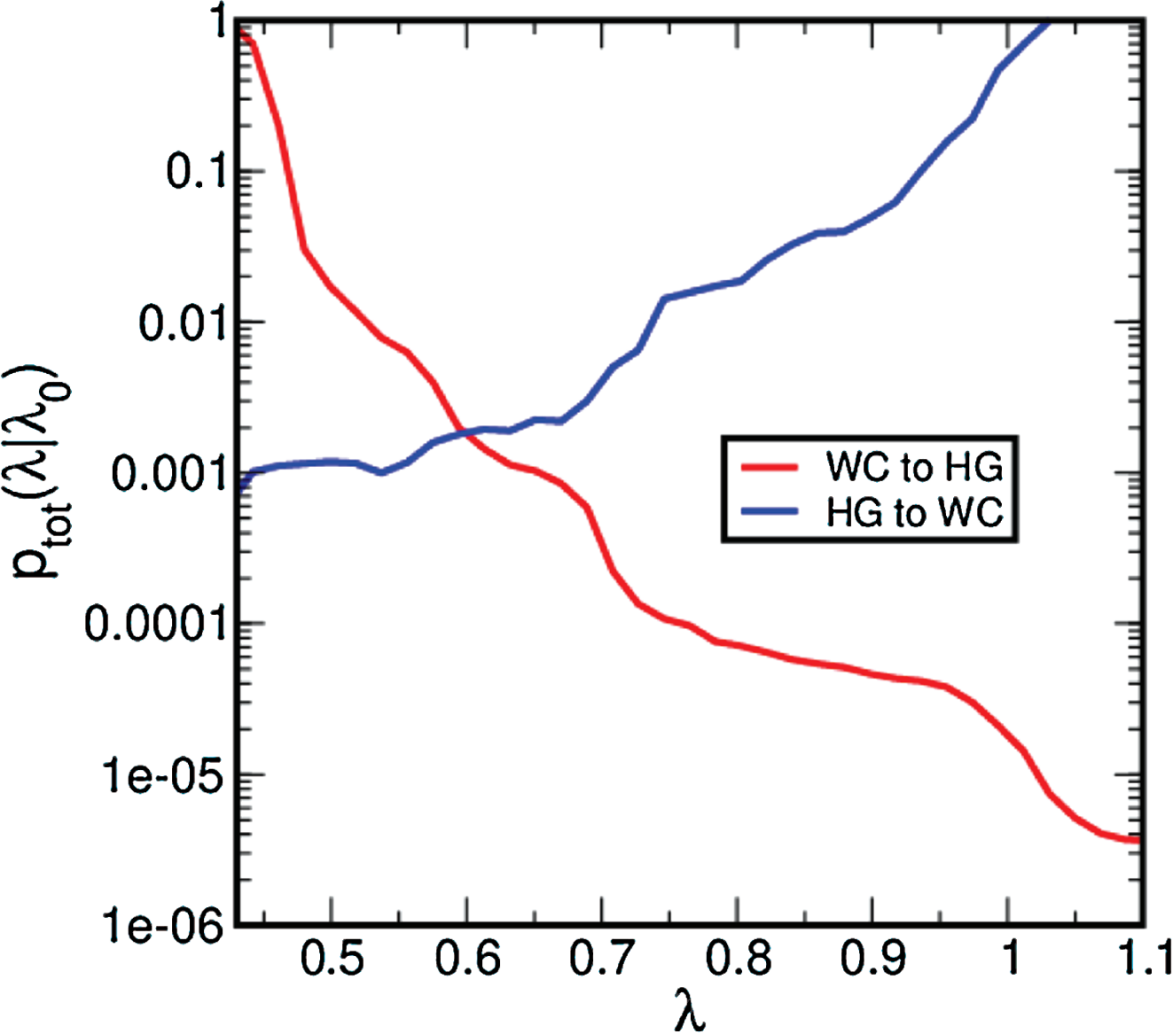
Total crossing probability for the WC to HG and HG to WC transitions as a function of λ. These curves are obtained by combining the crossing probabilities computed for each interface using the weighted histogram analysis method (33). More details are given in the Supporting Information.

To obtain the rate, we determined the crossing probability at λ = 1.0 for WC to HG, and at λ = 0.5 for HG to WC, as well as the probability to reach the HG or the WC state, respectively, see Figure 6. The slope of the log of the crossing probability *p*_tot_(λ_*i*_|λ_0_) as a function of λ for the WC to HG transition changes several times, indicating that even though it is possible to sample the WC to HG transition along the ${\rm arctan2}(d_{\rm {WC}},d_{\rm {HG}})$ order parameter, there are regions in which the system tends to stay longer. These regions occur where the slope is flatter, at λ < 0.5, λ = 0.7 and λ = 0.95. These regions correspond to the high density regions in the TPS path density plots, see Figure 4.

At λ = 1.0, *p*_tot_(λ_*i*_|λ_0_) = 1.5 × 10^−5^. Multiplying this with the probability to reach the HG state $p(\rm {HG}|\lambda _i)$ = 0.09 and by the flux out of the WC state Φ_WC_ = 5.5 × 10^8^ s^−1^, we computed the rate for the WC to HG transition to be $k_{\rm {WC}\rightarrow \rm {HG}}$= 742 s^−1^. Similarly we computed the rate for the HG to WC transition, using *p*_*tot*_(λ_*i*_|λ_0_) = 1.2 × 10^−3^ at λ = 0.5, the probability to reach the WC state $p(\rm {WC}|\lambda _i)$ = 0.94 and the flux out of the HG state Φ_HG_ = 1.45 × 10^9^ s^−1^, resulting in a rate of $k_{\rm {HG}\rightarrow \rm {WC}}$= 1.6 × 10^6^ s^−1^. Table 1 shows the rates as obtained from TIS simulations and from carbon dispersion relaxation NMR experiments (4). The rates computed by TIS are consistently 50 times faster than the experimentally determined rates. Taking the ratio of the two rates, which is the same as the Gibbs free energy difference between the WC and HG states, shows agreement between the computed and experimental rates.

**Table 1.** Rate constants

	experiment	TIS
$k_{\rm {WC}\rightarrow \rm {HG}}$ (s^−1^)	14.2 ± 1.03	742
$k_{\rm {HG}\rightarrow \rm {WC}}$ (s^−1^)	3670	1.6 × 10^5^
Δ*G* (k_B_T)	5.5	5.4

The free energy difference Δ*G* is calculated as the ratio of the rate constants $k_{\rm {WC}\rightarrow \rm {HG}}/k_{\rm {HG}\rightarrow \rm {WC}}$.

One explanation for the fact that the rates as computed by TIS are faster than the experimental data could be related to the force field we have used. To check this we performed TPS simulations of the *outside* transition with a force field containing improved parameters for nucleic acids, parmbsc1 (34); see the Supporting Information, in particular Supplementary Figure S8. These simulations give similar results as the *outTPS* simulations, indicating that the force field used in this study provides a sufficiently accurate description. The dihedral potential for the glycosidic angle is stiffer in the parmbsc1 force field, which results in higher barriers for the rotation along this bond, and may result in lower rates. Another issue with these force fields in general is the description of the interactions between the atoms. We have used a fixed-charge pairwise additive force field to keep the simulations with an aggregate simulation time of several μs tractable. However, this simplified description may have resulted in an overestimation of the rate constants.

## CONCLUSION

We have presented TPS simulations describing the transitions between the well-known Watson–Crick base-pairing motif and the Hoogsteen base-pairing motif. Recent experiments have shown that HG pairing, in which the purine is ‘upside-down’ relative to the WC motif, makes up a non-negligible fraction of DNA at physiological conditions. Using TPS, we obtained over 3500 samples of the transition between WC and HG, providing valuable insight into the mechanism of this transition. There are two main ways that the WC to HG transition can occur: either the purine stays in the double helix and rolls over directly, the *inside* route, or it goes *outside*, becomes exposed to solvent, rolls over, and re-enters the double helix. Thorough analysis of the relevant order parameters and a rough Bayesian analysis showed that this *outside* mechanism is preferred. We computed the rate constants for the WC to HG conversion and vice versa using TIS, as well as the relative free energies for the WC and HG states. Our results are in agreement with experimental data.

## Supplementary Material

gkz837_Supplemental_File

## Notes

Present address: David W.H. Swenson, Univ Lyon, ENS de Lyon, Univ Claude Bernard, CNRS, Laboratoire de Physique and Centre Blaise Pascal, F-69342 Lyon, France.
