# Supplementary material for: Atomistic insight into the kinetic pathways for Watson–Crick to Hoogsteen transitions in DNA
Source: Nucleic Acids Res. 2019 Oct 30;47(21):11069–76. doi: 10.1093/nar/gkz837 (PMC6868366; doi:10.1093/nar/gkz837)
Supplement: gkz837_Supplemental_File [file gkz837_supplemental_file.pdf]

Supplementary Information for

Atomistic insight into the kinetic pathways for Watson-Crick to Hoogsteen transitions in DNA

Jocelyne Vreede, Alberto Pérez de Alba Ortíz, Peter G. Bolhuis, and David W.H. Swenson

David W.H. Swenson.
E-mail: dwhs@hyperblazer.net

This PDF file includes:

Supplementary text
Figs. S1 to S12
Table S1
References for SI reference citations

Supporting Information Text

1. Structural aspects of base pairing

DNA is composed of a limited set of nucleotide building blocks that form specific hydrogen bonds, resulting in the base pairs AT and GC. These hydrogen bonds, together with van der Waals interactions between the nucleobases and hydrophobic interactions drive the formation of secondary and tertiary structure in DNA. In addition, negative charges on the phosphate group of each nucleotide cause repulsive forces within and between nucleic acids. In DNA, going from the Watson-Crick (WC) to Hoogsteen (HG) geometry, the flip of the purine (from *anti* in WC to *syn* in HG) is accomplished by a 180° rotation of the base along the bond connecting the base to the sugar, known as the glycosidic bond. Compared to the hydrogen bond scheme proposed by Watson and Crick, the bases are in closer proximity, requiring constriction of the DNA double helix by 2.5 Å (1). Molecular simulation studies revealed that the stability of Hoogsteen base pairing in DNA duplexes is comparable to that of WC pairing (2). For an AT pair, the difference in free energy between WC and HG is on the order of a hydrogen bond (12 kJ/mol) (3). The Hoogsteen conformation is significantly less energetically favorable for a CG pair, as the cytosine needs to be protonated (4), see Figure S1. Also, a GC^{+} pair in HG conformation lacks one hydrogen bond compared to a GC base pair in WC geometry. For AT no net change in number of hydrogen bonds occurs when converting from the WC to HG geometry, see Fig. S1 and Fig. 1 in the Main Text. In the AT base pair, a third non-canonical hydrogen bond may occur, between atom C2 in adenine and atom O2 in thymine, as indicated by QM studies on an AT base pair (5–7). This hydrogen bond can only occur in the WC state as the donor and acceptor are too far apart in the HG conformation. However, it may be possible for a non-canonical hydrogen bond to form between atoms C8 in adenine and atom O2 in thymine.

2. Computational details

System preparation. Using the make_na webtool (8) we created an ideal B-DNA duplex structure for nucleotide sequence 5'-CGATTTTTTGGC-3' (complementary strand 5'-GCTAAAAAACCG-3'), selected from Ref. (3). Nomenclature is according to the Protein Databank convention (9), with a prime indicating the complementary strand in the duplex. The structure was placed in a periodic dodecahedron box, followed by the addition of 6691 water molecules and 25 mM NaCl (6 Cl^{-} and 28 Na^{+} ions), resulting in a charge-neutral system of 20868 atoms. The concentration of 25 mM NaCl was chosen to mimic the experimental conditions of Ref. (3) Preparation of the system consisted of energy minimization (conjugate gradient with a force threshold of 100 kJ/mol nm) and a 1 ns position restrained molecular dynamics (MD) run, with restraints on the DNA heavy atoms using a force constant of 1000 kJ/mol nm in each direction. We used the AMBER03 force field (10) to describe the interactions between atoms, in combination with the TIP3P water model (11). Non bonded interactions were treated with a cut-off at 0.8 nm, and long range electrostatics were handled by the Particle Mesh Ewald method (12, 13). After equilibration, nine 200 ns molecular dynamics runs at constant temperature and pressure were initiated with different random inital velocities taken from the Maxwell-Boltzmann distribution at 300 K. The v-rescale thermostat (14) kept the temperature constant at 300 K and the Parrinello-Rahman barostat (15) kept the pressure constant at 1 bar. All molecular dynamics simulations were performed with GROMACS version 4.5.3 (16).

Calculation of order parameters. In the WC state, see Figure S1, a hydrogen bond is formed between atom N3 in residue DT9 and atom N1 in residue 4DA', while in the HG state atom N3 in residue DT9 and atom N7 in residue 4DA' form the hydrogen bond. A hydrogen bond between atoms O4 in residue DT9 and N6 in 4DA' occurs in both states, indicated as BP. A hydrogen bond is considered formed if the distance between the donor and acceptor is less than 0.35 nm and the angle between the acceptor, donor and hydrogen atom is less than 30°. To determine whether hydrogen bonds are formed between the bases we calculated the distances d_{BP}, between atom O4 in residue DT9 and atom N6 in residue 4DA', distance d_{WC}, between atom N3 in residue DT9 and atom N1 in residue 4DA' and distance d_{HG}, between atom N3 in residue DT9 and atom N7 in residue 4DA'. The labels d_{BP}, d_{WC} and d_{HG} indicate the distance between the atoms involved in the hydrogen bond that is always formed, the hydrogen bond that is formed in the Watson-Crick state and the hydrogen bond that is formed in the Hoogsteen state, respectively. The hydrogen bond distances d_{WC} and d_{HG} can be plotted as a single coordinate in the form of $\lambda = \arctan 2(d_{\mathrm{WC}}, d_{\mathrm{HG}})$. The conversion from WC to HG in the d_{WC}, d_{HG} plane can be considered as traversing one quadrant of a circle and the position on that circle provides adequate description of the transition progress, with λ is 0.46 and and 1.11 for WC and HG respectively.

In addition we calculated the glycosidic angle χ, the base opening angle θ and the base rolling angle ϕ, see Fig. 3 in the Main Text. The glycosidic angle χ is the dihedral angle involving atoms O4', C1', N9, C4 in residue 4DA'. The base opening angle θ (17) is defined as the angle between two planes: the first spanned by the center of mass of the adjacent base pairs (8DT-5DA' and 10DG-3DC'), and the center of mass of the phosphate groups in residues 4DA' and 5DA', and the second plane defined by the center of mass of the base of residue 4DA' and again the center of mass of the phosphate groups in residues 4DA' and 5DA'. The base rolling angle ϕ is defined as the angle between two vectors: one vector *bb* connects the phosphorus atoms in residues 11DG and 11DC', which is a proxy for the long axis of the DNA duplex, and the other vector *bp* is the normal to the plane of the base. The plane of the base is defined by the vectors connecting atoms N3 and N1 and N3 and N7 in residue 4DA', the rolling base. See Fig. 3 in the Main Text for a schematic representation of these three angles.

We used GROMACS tools (16) to calculate these parameters in combination with Perl and Python scripts to calculate the base opening and rolling angles.

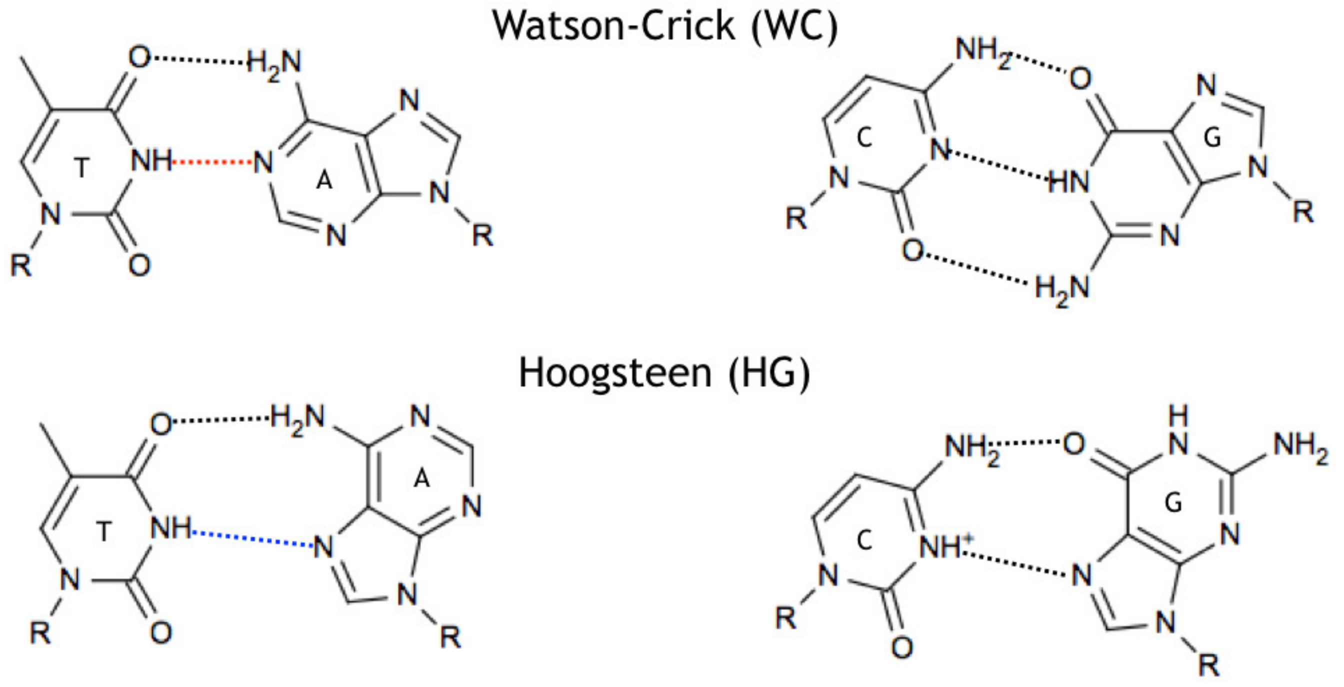

Fig. S1. Hydrogen bond patterns for thymine (T) - adenine (A) (left) and cytosine (C) - guanine (G) (right) base pairs in Watson-Crick (WC) (top) and Hoogsteen (HG) (bottom) configuration.

Metadynamics. The structure obtained after the equilibration procedure was also used as a starting point for several metadynamics simulations (18) aimed at generating an initial transition path for use in the path sampling simulations. The collective variables used in these simulations were the glycosidic angle χ and the distance between atom O2 in residue DT9 and atom N7 in residue 4DA'. We used hills with a height of 0.25 kJ/mol and a width of 0.35 rad for the dihedral and 0.1 nm for the distance, with various deposition rates. Note that we did not intend to converge these metadynamics simulations, but instead, we were aiming to obtain an initial reactive pathway. The metadynamics simulation biasing on χ and the distance with a deposition rate of 2 ps^{-1} resulted in an *outside* WC to HG transition. Another metadynamics runs biasing on the dihedral angle connecting the adenine to the deoxyribose in residue 4DA' and a deposition rate of 2 ps^{-1} resulted in several WC to HG transitions occurring via the *inside* route. See Fig. S2 for time traces of the hydrogen bond distances d_{WC}, d_{HG} and d_{BP} during both metadynamics simulations. In addition, the metadynamics simulation resulted in several HG conformations, of which we used one as a starting conformation for five 200ns MD simulations. All metadynamics simulations were performed with PLUMED version 1.2.2 (19).

Transition path sampling. Transition path sampling (TPS) samples reactive pathways connecting two stable states employing a Metropolis Monte Carlo scheme (20, 21). Initializing a TPS simulation requires order parameter based definitions for the stable states and an initial reactive trajectory containing positions and velocities, connecting the two stable states (22). This trajectory does not need to be an equilibrium trajectory, as TPS allows for equilibration of the pathways to specific conditions. As stable state definitions we used the differences in hydrogen bond pattern between the Watson-Crick and Hoogsteen states. The system is considered to be in the WC state if both distances d_{BP} and d_{WC} are below 0.35 nm, while the system is considered to be in the HG state if both distances d_{BP} and d_{HG} are below 0.35 nm. The cut-off of 0.35 nm is based on the maximum distance between a hydrogen bond donor and acceptor in a hydrogen bond.

We used in-house Perl scripts to perform the path sampling simulations. In total we performed two different sets of path sampling simulations: *outTPS* and *inTPS*. The *outTPS* simulations started from a transition with the adenine in residue 4DA' going outside the double helix into the solvent, a route referred to as *outside* and the *inTPS* simulations started from a transition with the adenine remaining inside the double helix, referred to as *inside*. In addition, we performed a TPS simulation started from an *outside* path using the AMBER99-ILDN parmbsc1 force field (23). All TPS simulations were performed with the one-way shooting algorithm using randomly chosen shooting points on the reactive paths. We allowed for flexible lengths of the paths (22). Trajectories were monitored every 5 ps to see if they reached a stable state. To remove the biased dynamics, we performed a preparatory TPS simulation for each initial path. During this TPS simulation several cycles were performed, which consist of randomly selecting a frame from the last accepted trajectory, called 'shooting point', followed by propagation either forward or backward in time using straightforward MD. Note that the MD used for the propagation of the shooting point does not contain any biasing potentials. Equilibration of the initial paths was complete when the paths no longer shared any frame with the original metadynamics paths, meaning that they are generated without the aid of any biasing potential. Equilibration of the metadynamics pathway occurred within 10 accepted paths. The equilibrated path was then used as the starting path for 10 independent transition path sampling runs for each set of simulations. We used the same settings for the MD runs used in the TPS as described above, with the v-rescale thermostat (14) introducing stochasticity allowing divergence of the paths.

Path length distributions were calculated making a histogram over the length of each path. The path density plots show the fraction of pathways in the TPS ensemble that pass through the given values of the order parameters at least once (22). These plots were prepared as follows. Each pathway in the ensemble was smoothed by taking a running average with a window of 100 ps. Next, by discretizing each of the order parameter intervals in 100 bins, we constructed for each trajectory in the ensemble a binary matrix, in which 1 means that the path visits the bin at least once, and 0 means no visitations at all. These matrices were subsequently ensemble-averaged, resulting in path density maps.

Transition Interface Sampling. Rate constants can be computed with Transition Interface Sampling (TIS) (24). Like TPS, this method is a Monte Carlo procedure in path space, sampling multiple path ensembles. Each path ensemble involves an 'interface', or hypersurface for a fixed value of a predefined order parameter λ, which should be a reasonable approximation of the progress of the transition. Considering a two-state system with states A and B, the paths in the ensemble for interface A_i begin in state A, must cross the hypersurface at λ_i, and can end in either state A or state B (where i is the index of the interface). The restriction that all paths in interface A_i must cross λ_i means that sampling such paths allows for the calculation of $p_A(\lambda_{i+1}|\lambda_i)$, the probability that a path crosses the next interface given that it crosses A_i. The rate in TIS is given by

$$k_{AB}^{\mathrm{TIS}} = \Phi_A \prod_{i=0}^{m-1} p_A(\lambda_{i+1}|\lambda_i) p_A(B|\lambda_m) \qquad [1]$$

where Φ_A is the flux from state A through its innermost interface, and $p_A(B|\lambda_m)$ is the probability that a trajectory in the outermost interface from state A ends in state B. This description is based on the two-state version of TIS; it has also been extended to multiple state systems (25, 26). In practice, the product over successive crossing crossing probabilities can be calculated more accurately using the weighted histogram analysis method (WHAM) (27) to combine the histograms, creating a total crossing probability function $P_A(\lambda|\lambda_0)$ that can be evaluated at $\lambda = \lambda_m$.

As an order parameter we used $\lambda = \arctan 2(d_{\mathrm{WC}}, d_{\mathrm{HG}})$ to sample the WC to HG transition as well as the HG to WC transition, using 14 and 13 interfaces respectively. Positioning of the interfaces was such as to ensure crossing probabilities of

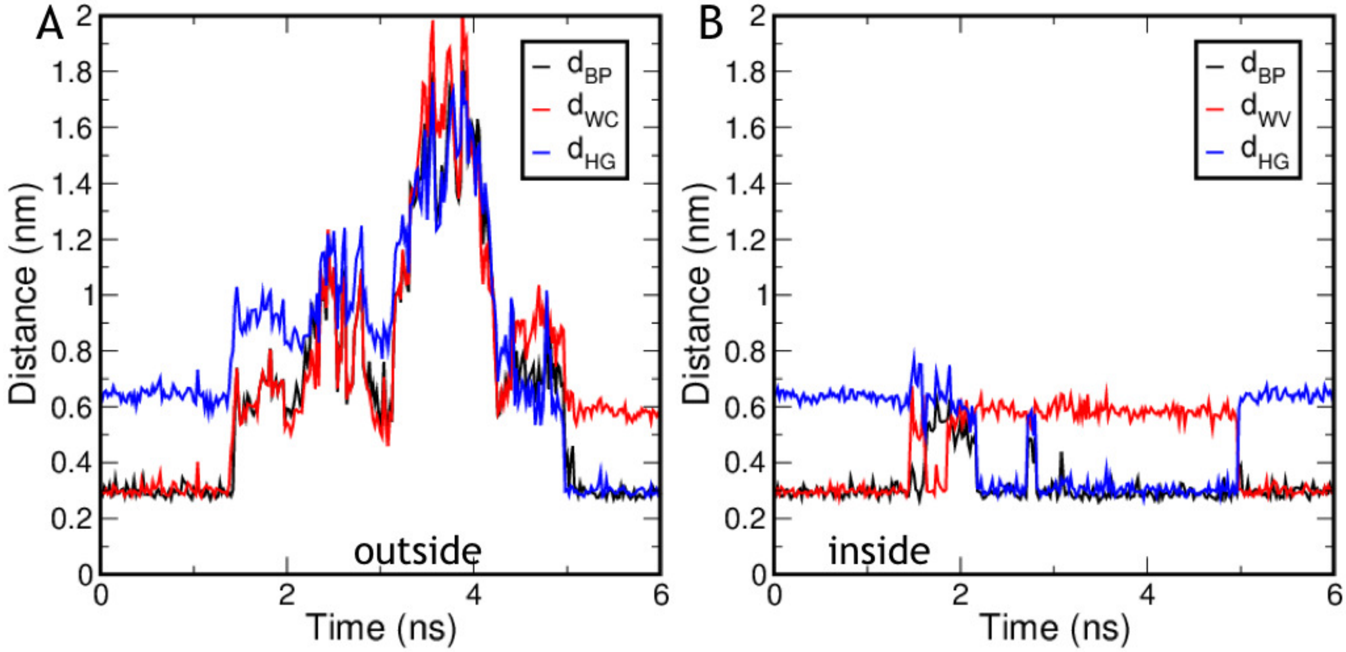

Fig. S2. Time traces of the three specific hydrogen bonds in two metadynamics simulations. Panel (A) shows an outside transition and panel (B) shows an inside transition.

at least 10%. The interfaces were located at $\lambda = 0.5$, 0.55, 0.6, 0.65, 0.7, 0.725, 0.75, 0.8, 0.85, 0.9, 0.95, 1.0. For the WC to HG transition two additional interfaces at $\lambda = 0.43$ and $\lambda = 0.48$ were added. For the HG to WC transition an extra interface was added at $\lambda = 1.05$. For each interface we generated at least 500 accepted paths, of which the first 60 were not included in further calculations and analyses to allow for equilibration. For each interface we then determined the crossing probabilities, which were combined into the total crossing probability using WHAM (27) as implemented in OpenPathSampling (28).

Calculation of the flux. For the WC and HG states, the flux out of the state and through the first interface was calculated from respectively nine and five times 200 ns MD simulations. Trajectories were considered to have crossed the innermost interface when $\lambda > 0.45$ for WC, and when $\lambda < 1.02$ for HG. The flux is given by $\Phi = (\langle t^- \rangle + \langle t^+ \rangle)^{-1}$, where t^- is the time from when a trajectory segment enters the interface until it next exits the state, and t^+ is the time from when the segment exits the state until it next enters the interface (29). This analysis was performed with OpenPathSampling (28). The resulting values for the flux out of the WC and HG states are $\Phi_{\mathrm{WC}} = 5.5 \cdot 10^8$ s^{-1} and $\Phi_{\mathrm{HG}} = 1.45 \cdot 10^9$ s^{-1}. Note that the definitions for the stable states in this calculation are slightly different than as used in the TPS and TIS simulations, as we used the atan2 function instead of the actual distances.

3. Choice of order parameters

The glycosidic angle χ in the residue that undergoes the WC to HG transition has distinct values specific for each state, as shown in Fig. S3. For WC χ ranges between -170° and -45°, and for HG χ ranges between -45° and 60°. During 200 ns of MD χ jumps between these two ranges and stays within one region for several tens of ns. Visual inspection of the trajectories showed that even though the base remains paired to its partner through hydrogen bonds, several conformational rearrangements occur in the DNA backbone, involving the deoxyribose group. Specifically, fluctuations in the phosphodiester backbone torsion angles can change the orientation of the deoxyribose group and cause jumps in the glycosidic angle χ. However, these jumps are not due to the rolling of the adenine—which remains aligned and even H-bonded with the thymine—but to the rolling of the sugar. In addition, variations in the sugar ring torsion angles, i.e., sugar puckering, can influence the position of the O4' atom and also induce alterations in the glycosidic angle χ. Such deformations near the glycosidic bond do not only occur in the stable base-pairing states, but also during transitions. It is highly non-trivial to combine the phosphodiester backbone, sugar and glycosidic angles into a single order parameter to describe the rotation of the adenine precisely. This motivates the definition of an order parameter independent of local deformations, similar to the one in Ref. (30). To obtain a better discrimination of WC and HG based on the orientation of the base, we calculated the base rolling angle ϕ which is 20° for WC and -135° for HG, but can extend to -180° and then to 180°. No jumps or large fluctuations occur in this parameter. To illustrate this, Fig. S3 shows time traces for the angles χ and ϕ for the same simulation data, with ϕ exhibiting smaller fluctuations and no jumps.

4. Results TPS simulations

We performed 30 TPS simulations, 10 starting from an inside path, called *inTPS*, 10 starting from an outside path, called *outTPS*, and 10 runs starting from an outside path using the parmbsc1 force field. For the AMBER-03 runs we performed at least 500 trial moves. For each parmbsc1 runs we collected 100 accepted paths, which required a total of 3318 trial moves for all ten runs. Statistics for each type of TPS run are listed in Table S1. Fig. S4 shows the path length distribution and Fig. S5 displays an example of a sampling tree, taken from an *outTPS* run.

Distinguishing inside and outside paths. The base opening angle θ allows for a way to distinguish between *inside* and *outside* paths. In the *outside* route, the adenine residue undergoing the transition becomes exposed to solvent. Including the number of water molecules around relevant atoms involved in the transition may provide a better way to determine when a path takes the *outside* or the *inside* channel. To determine which calculation of the number of waters gives the best separation of the two channels, in combination with θ, we computed path density profiles for the number of water molecules around three sets of atoms: all non-hydrogen atoms in the rolling base 4DA', atom N6 in rolling base 4DA' and atom N3 in the base opposite the rolling base DT9. We also checked the effect of counting the number of water molecules in different radii around these sets of atoms, by counting the number of water oxygen atoms within a radius of 0.3 nm, 0.4 nm, 0.5 nm and 0.6 nm. These are shown in Fig. S6. A radius of 0.6 nm already gives a clear separation of *inside* and *outside* paths.

Defining the boundary between inside and outside. Plotting θ_{min} and the maximum value for $N_{\mathrm{water,max}}$ suggests that there are two sets of paths which overlap. These sets can be separated by a linear function $N_{\mathrm{water,max}} = 0.185\theta_{\mathrm{min}} + 32$, as indicated by the red line in Fig. 5C in the Main Text. Paths below this line are *inside* paths, and paths that are above the dividing line are *outside* paths. This dividing line was fitted to two points in the region of the lowest density in between *inside* and *outside*. To check if this selection criterion indeed results in a separation of *inside* and *outside* paths, we plotted path density profiles for both sets in the λ, θ and the λ, ϕ planes. These sets do not include the paths classified as neither *inside* nor*outside*. The path density plots show that the *inside* set did not contain any *outside* paths, but the *outside* set did contain *inside* paths. The separation improved tremendously when we introduced a region in which paths are classified as neither *inside* nor *outside* with a margin of $N_{\mathrm{water,max}} = 2$ above and below the dividing line. The resulting path density profiles are shown in Fig. S7. The *inside* set does not contain any *outside* path, but the *outside* set still contains *inside* paths. When extending the margin up to 10 water molecules, the *outside* set still contains *inside* paths. We therefore selected a margin of 2 water molecules to include a larger number of paths within the *inside* and *outside* sets. The one-way shooting algorithm could be a possible explanation for

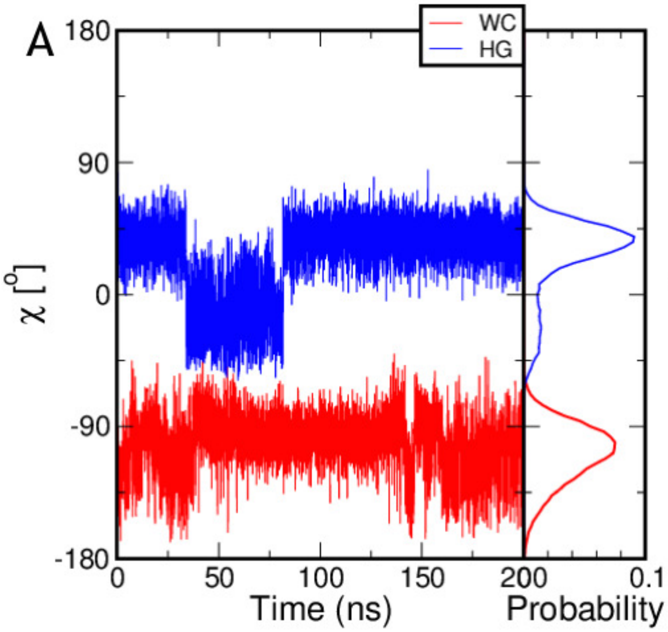

Fig. S3. Time traces and probability histograms of the glycosidic angle χ (A) and the base rolling angle ϕ (B) for the MD simulations of WC (red) and HG (blue). The time traces are taken from the same simulations. The probability histograms are calculated over all MD data.

Table S1. TPS statistics

	outside	inside	parmbsc1
number of accepted paths	1716	1880	1000
acceptance ratio	0.28	0.31	0.30
number of decorrelated paths	125	124	15

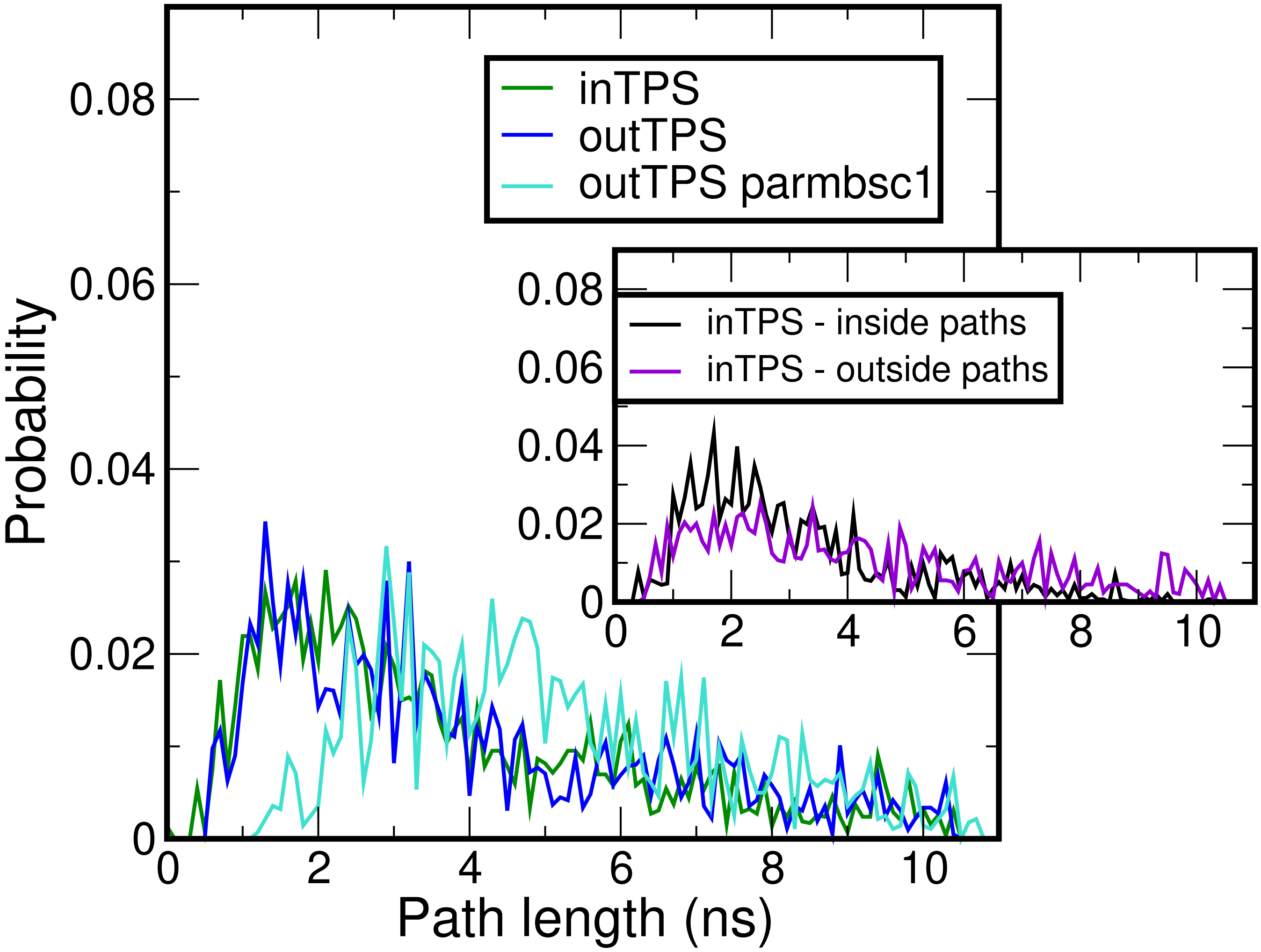

Fig. S4. Distribution of the path length. The distribution is calculated by computing a histogram of the path length for each accepted path, multiplied by its path weight.

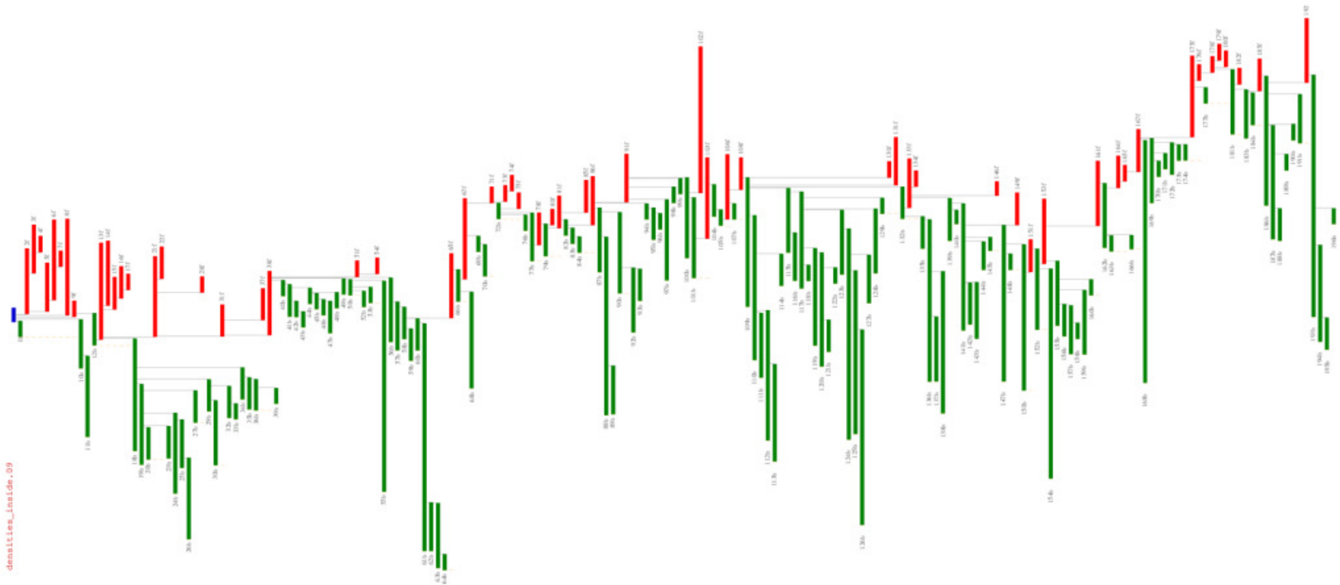

Fig. S5. Example of a sampling tree, taken from an *outTPS* run. The blue bar indicates the starting path. The red and green bars indicate accepted path going forward (to HG) and backward (to WC) respectively. The vertical black lines indicate from which path the shooting point originates.

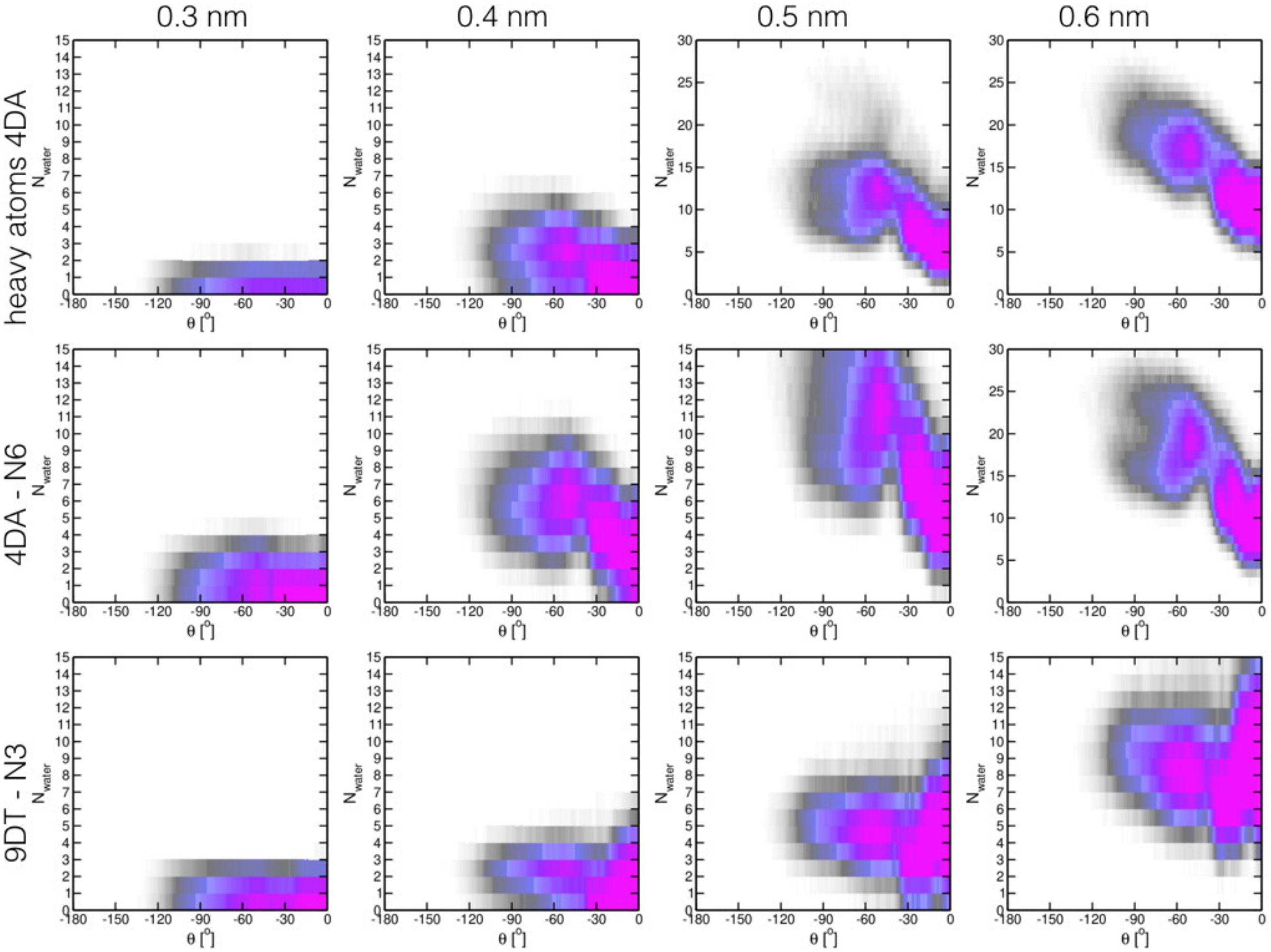

Fig. S6. Path density plots in the θ,$N_{\rm water}$ plane for different definitions of $N_{\rm water}$. The color of the path density ranges from white (no paths) to magenta (highest path density).

the occurrance of *inside* paths in the *outside* selection as paths that start out as *inside* can switch to *outside* while retaining some of the *inside* character.

The parmbsc1 force field gives similar results. We performed all these simulations with the AMBER03 force field, for which the interactions in nucleic acids are not well described. The parmbsc1 parameter set contains optimized parameters for bond rotations in nucleic acids (23). We performed path sampling simulations of the *outside* transition by first forcing the transition in a metadynamics run using a path variable as described in Ref. (30).

This simulation resulted in an *outside* transition, which we equilibrated to the unbiased dynamics settings using path sampling. The equilibrated path was then used to start ten independent path sampling simulations, resulting in 2017 accepted paths with an average length of 3 ns, see Figure S4. Table S1 gives the details of the TPS simulations. The number of decorrelated paths is much lower for the parmbsc1 simulations compared to the TPS simulations using the AMBER-03 force field. One of the main differences between the two force fields is that the dihedral potential for the glycosidic bond is much stiffer in parmbsc1, thus increasing the barrier for rotation (23). A higher rotational barrier for the glycosidic bond would result in less decorrelated paths given a similar number of trials in the TPS simulations. The stiffer potential for the glycosidic rotation also explains why the average path length is longer for the parmbsc1 simulations, see Fig. S4. Fig. S8 shows the path density in the (λ, ϕ) and (λ, θ) planes. These path densities look similar to the path densities observed for the *outTPS* simulations in the Main Text, Fig. 4.

5. Results TIS simulations

As the TPS results indicate that the *outside* route is the dominant mechanism for the WC to HG conversion, all interfaces are initialized from an *outside* path. The path density as a function of λ, the base opening angle θ and the base rolling angle ϕ for each interface are shown in Figs. S9-S12. For the WC to HG transition, the path density for the interfaces at $\lambda < 0.5$ occurs at $\theta >$ -32°. The interfaces at $\lambda > 0.8$ all show path density for $\theta <$ -32°. The interfaces at λ between 0.55 and 0.75 show density at θ both close to zero and far from zero. These observations indicate that interfaces close to the final state sample *outside* transitions. Interfaces close to the initial states do not sample such large ranges for θ, indicating that the distinction between *inside* and *outside* becomes relevant only after a certain progression of the transition. The path density profiles suggest that the choice for going via the *inside* or the *outside* channel lies at $0.65 < \lambda < 0.75$.

6. Bayesian analysis

Given two channels *outside* and *inside*, we define the equilibrium probabilities $P(\mathrm{TP_{in}})$ and $P(\mathrm{TP_{out}})$ for finding a transition path in the *inside* and *outside* channels respectively. These two probabilities are related by the detailed-balance-like relation

$$P(\mathrm{outside} \rightarrow \mathrm{inside}|\mathrm{TP_{out}})]P(\mathrm{TP_{out}}) = \\ P(\mathrm{inside} \rightarrow \mathrm{outside}|\mathrm{TP_{in}})P(\mathrm{TP_{in}}),$$

where $P(\mathrm{inside} \rightarrow \mathrm{outside}|\mathrm{TP_{in}})$ is the probability that, given a path $\mathrm{TP_{in}}$ in the *inside* channel, the path sampling switches to the *outside* channel after a fixed amount of trial moves in a TPS run, and $P(\mathrm{outside} \rightarrow \mathrm{inside}|\mathrm{TP_{out}})$ the other way around. This simple statement says that the probability to start with an *outside* path and switching to an *inside* path is identical to starting with an *inside* path and switching to the *outside* channel. In other words, the switching rates between *outside* and *inside* are identical in an equilibrium path simulation. This requires the assumption that the channels are sufficiently sampled during the fixed length of the TPS run (500 MC trial moves in this case). We remark that this is similar in spirit to work by Stelzl and Hummer (31). Since we are interested in determining the relative probability $P(\mathrm{TP_{out}})/P(\mathrm{TP_{in}})$, i.e. the propensity to be on a *outside* path compared to an *inside* path, we can write

$$\frac{P(\mathrm{TP_{out}})}{P(\mathrm{TP_{in}})} = \frac{P(\mathrm{inside} \rightarrow \mathrm{outside}|\mathrm{TP_{in}})}{P(\mathrm{outside} \rightarrow \mathrm{inside}|\mathrm{TP_{out}})}. \quad [2]$$

That is, the equilibrium ratio of *inside* versus *outside* paths is identical to the ratio of the switching probability from *outside* to *inside* with respect to the switching probability from *inside* to *outside*.

To support the hypothesis that an *outside* path is more likely than an *inside* path we need to show that

$$P(\mathrm{inside} \rightarrow \mathrm{outside}|\mathrm{TP_{in}}) > P(\mathrm{outside} \rightarrow \mathrm{inside}|\mathrm{TP_{out}})$$

We need to provide evidence that this is true based on the measurements that we have from the TPS simulations. Defining the exact true value for $P(\mathrm{inside} \rightarrow \mathrm{outside}|\mathrm{TP_{in}}) \equiv \theta$ and $P(\mathrm{outside} \rightarrow \mathrm{inside}|\mathrm{TP_{out}}) \equiv \phi$, respectively, we need to show that

$$\theta > \phi.$$

However we do not have an exact value, only an approximate estimated value $\theta_{\mathrm{est}} = r_\theta/n$ and $\phi_{\mathrm{est}} = r_\phi/n$, where r_θ , and r_ϕ denote the number of realisations that an *inside* path switches to *outside*, and the number of realisations an *outside* path switching to *inside* respectively.

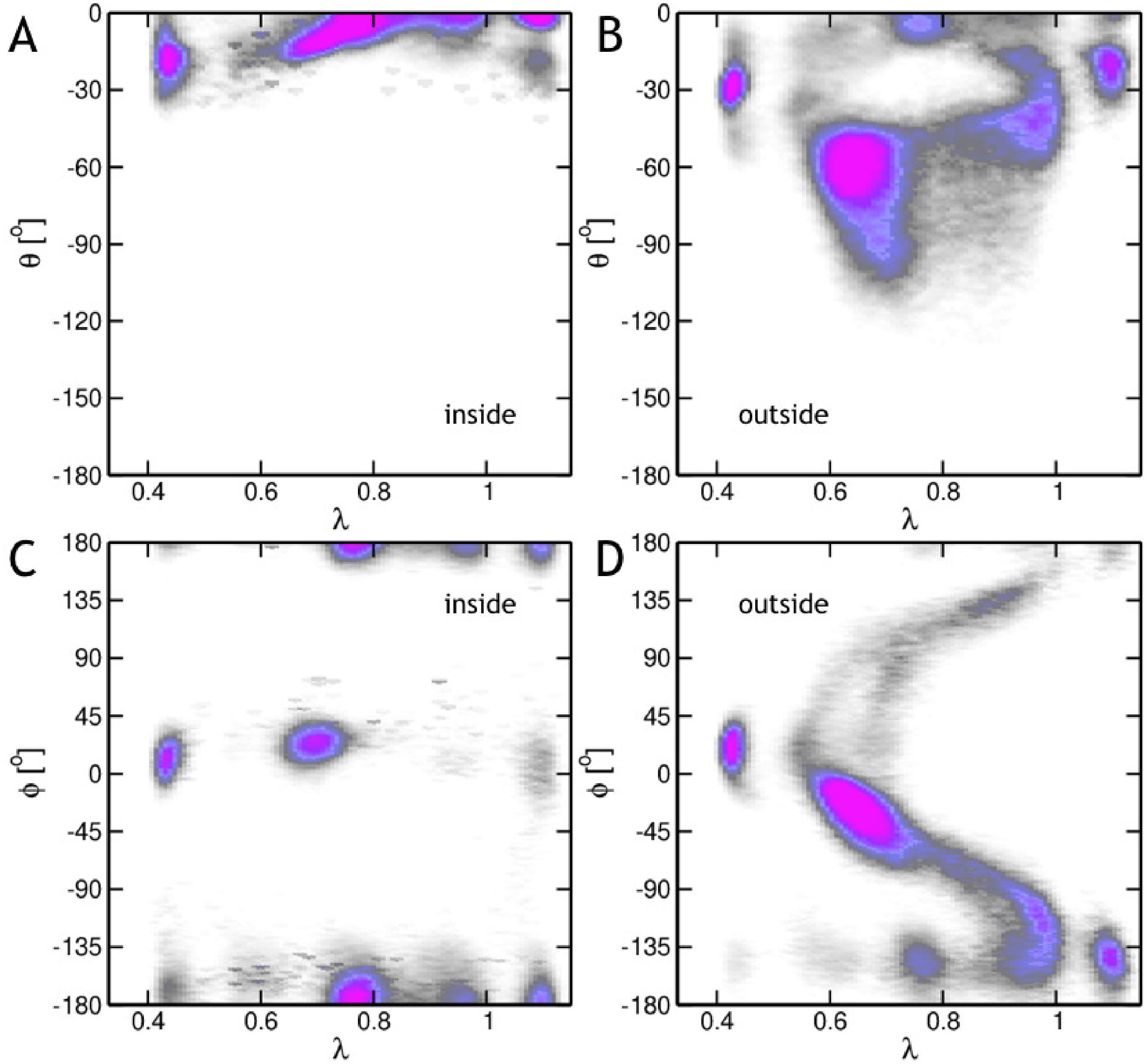

Fig. S7. Path density plots in the λ, θ plane (A, B) and the λ, ϕ plane (C,D) for (A,C) *inside* and (B,D) *outside* simulations. The color of the path density ranges from white (no paths) to magenta (highest path density).

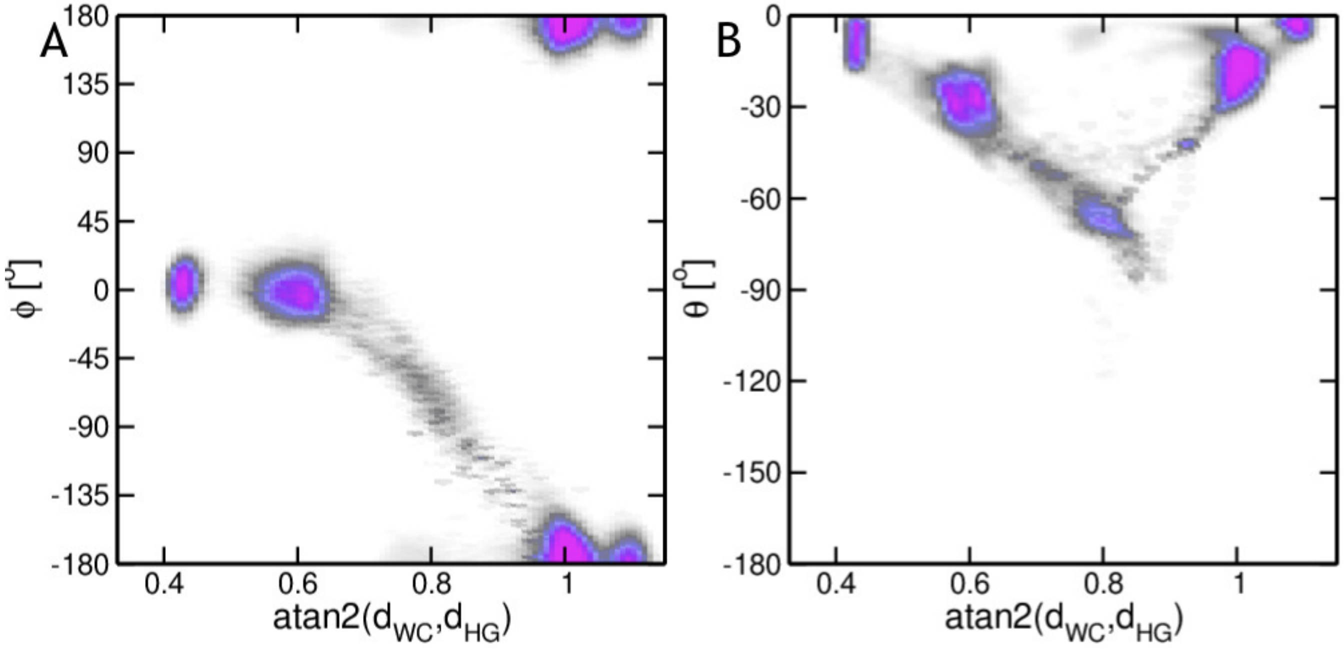

Fig. S8. Path density plots of the TPS simulation using the parmbsc1 force field in (A) (λ, ϕ) and (B) (λ, θ) projections. The color of the path density ranges from white (no paths) to magenta (highest path density).

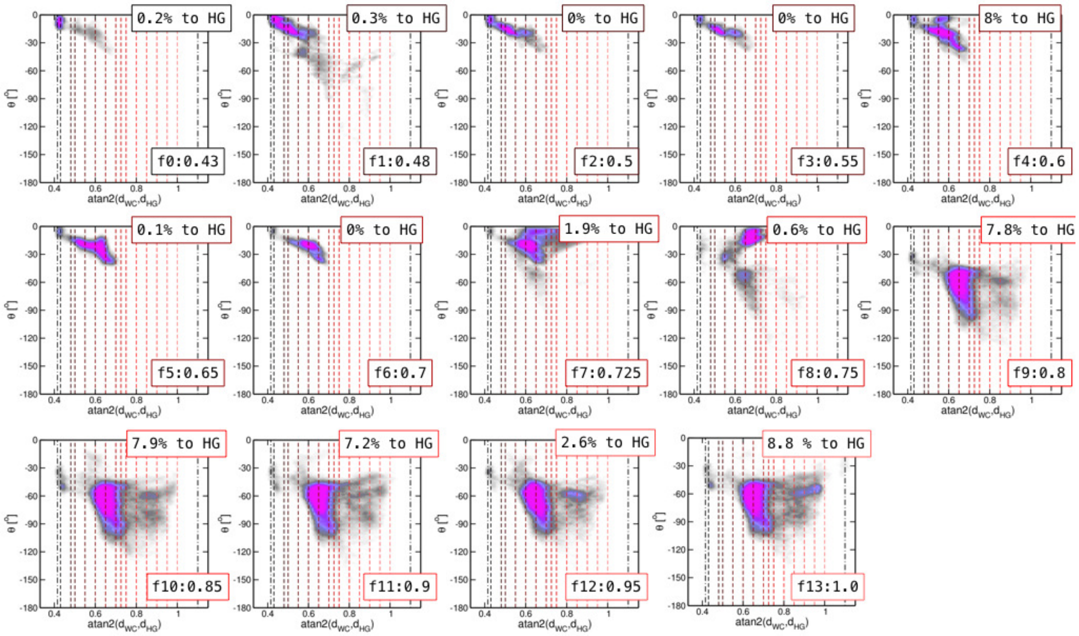

Fig. S9. Path density plots in the λ,θ plane for each interface in the WC to HG transition. The color of the path density ranges from white (no paths) to magenta (highest path density).The position of the interfaces in λ are shown as dashed lines.

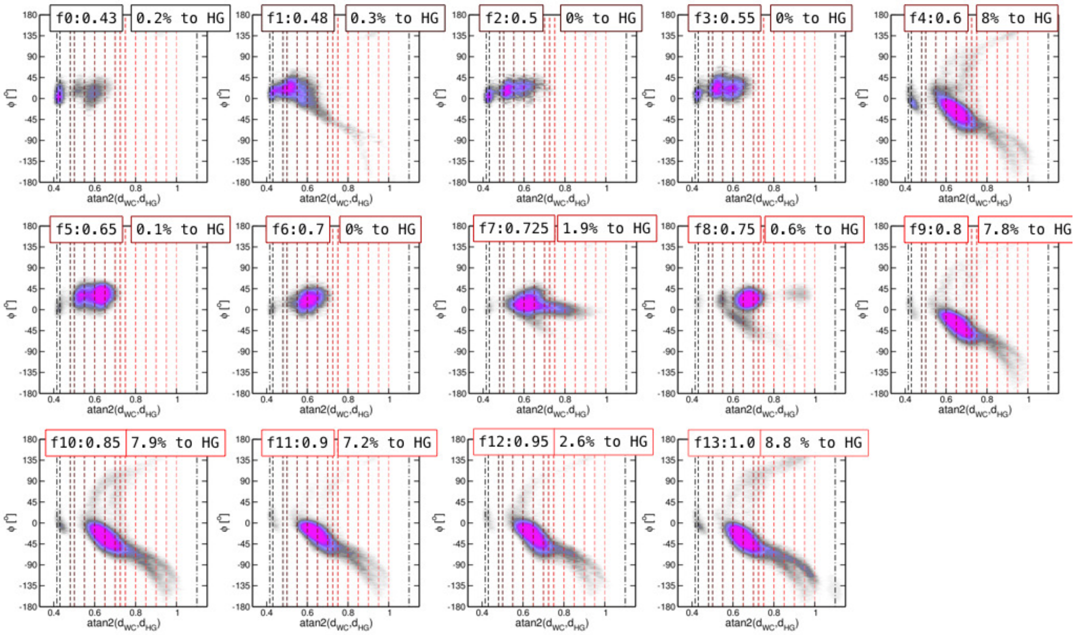

Fig. S10. Path density plots in the λ,ϕ plane for each interface in the WC to HG transition. The color of the path density ranges from white (no paths) to magenta (highest path density).The position of the interfaces in λ are shown as dashed lines.

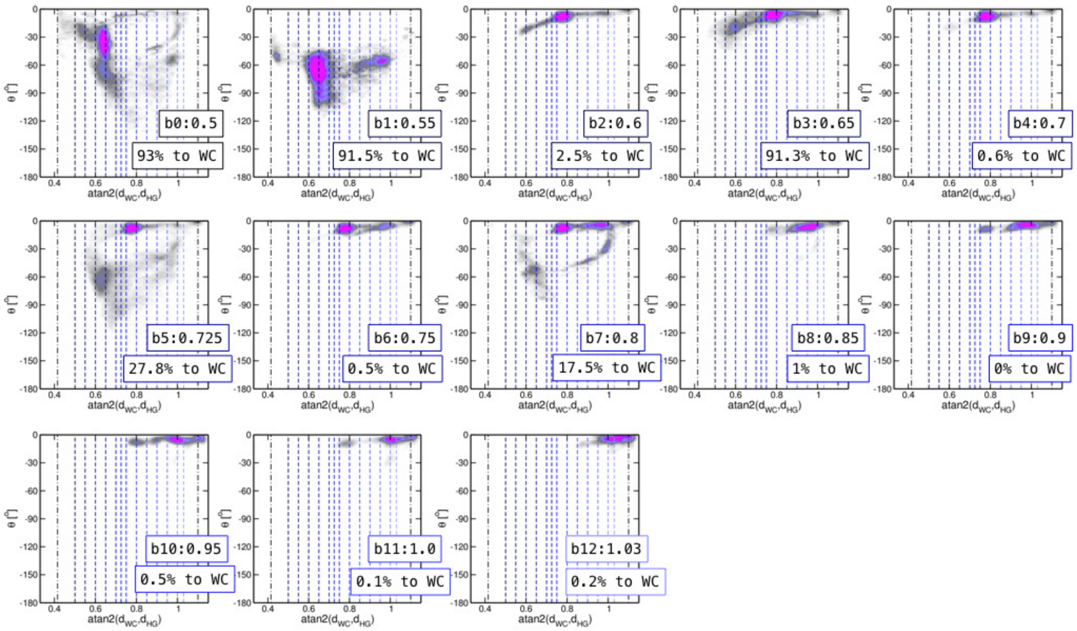

Fig. S11. Path density plots in the λ,θ plane for each interface in the HG to WC transition. The color of the path density ranges from white (no paths) to magenta (highest path density).The position of the interfaces in λ are shown as dashed lines.

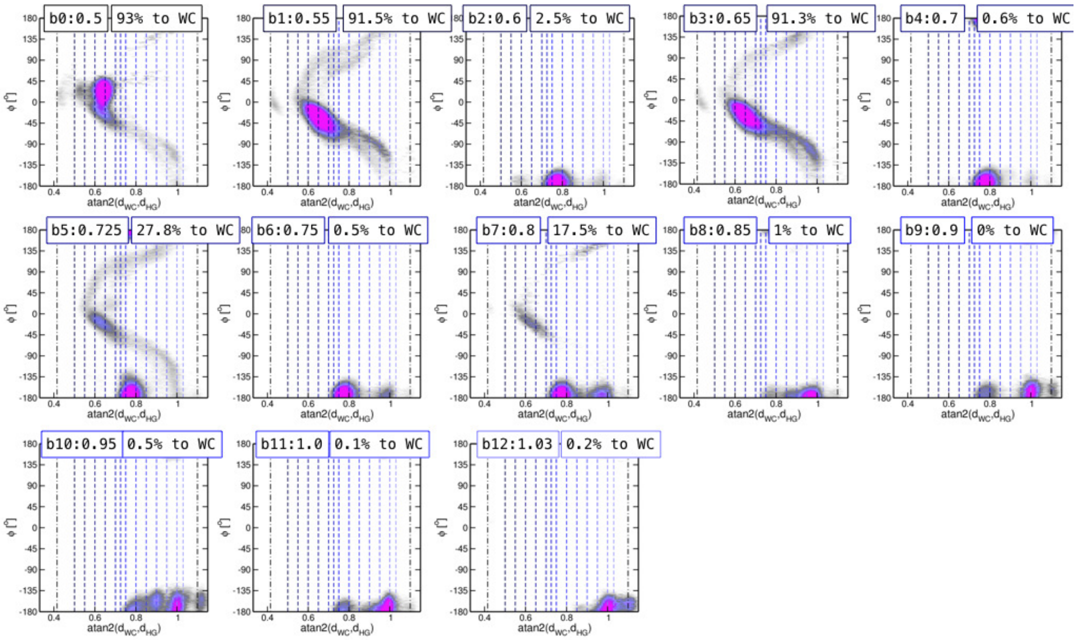

Fig. S12. Path density plots in the λ,ϕ plane for each interface in the HG to WC transition. The color of the path density ranges from white (no paths) to magenta (highest path density).The position of the interfaces in λ are shown as dashed lines.

Using standard Bayesian parameter estimation theory the probability of observing the data $D = \{r_\theta, n\}$, given a θ and prior information I, is determined by the binomial distribution

$$P(D|\theta I) = \theta^{r_\theta}(1-\theta)^{n-r_\theta}, \tag{3}$$

where r_θ is the number of switches from *inside* to *outside*, while $n - r_\theta$ is the number of times the trial path remains in the *inside* channel after a TPS move. Then using

$$p(DI)p(\theta|DI) = p(\theta|I)p(D|\theta I), \tag{4}$$

and realising that $p(DI)$ is a constant independent of θ, it follows that posterior probability distribution for θ is

$$p(\theta|DI) = \frac{p(\theta|I)p(D|\theta I)}{\int d\theta p(\theta|I)p(D|\theta I)}. \tag{5}$$

Assuming a uniform prior $p(\theta|I) = 1$ we achieve the posterior probability distribution for θ based on the data is

$$P(\theta|r_\theta, n) = A_\theta \theta^{r_\theta}(1-\theta)^{n-r_\theta}, \tag{6}$$

where we dropped the implicit dependence on the prior information I. Likewise for ϕ

$$P(\phi|r_\phi, n) = A_\phi \phi^{r_\phi}(1-\phi)^{n-r_\phi}. \tag{7}$$

Here, the A variables are normalisation constants, given by

$$A_\theta = \frac{(n+1)!}{(n-r_\theta)!r_\theta!} \tag{8}$$

$$A_\phi = \frac{(n+1)!}{(n-r_\phi)!r_\phi!}. \tag{9}$$

The probability that $\phi < \theta$ is then given by the integral over the joint distribution function $P(\theta, \phi|D) = P(\theta|r_\theta, n)P(\phi|r_\phi, n)$ for the area for which holds $\phi < \theta$. In a 2D representation with θ as the x-axis and ϕ on the y-axis this amounts to taking the integral under the diagonal $\phi = \theta$

$$P(\phi < \theta|D) = \int_0^1 d\theta \int_0^\theta d\phi P(\theta|r_\theta, n))P(\phi|r_\phi, n)), \tag{10}$$

where the data $D = \{r_\theta, r_\phi, n\}$ To make progress we set $r_\phi = 0$, as the number of observed switches from *outside* to *inside* equals zero. This means that

$$P(\phi|0, n) = (n+1)(1-\phi)^n. \tag{11}$$

Substituting this into Eq.10 gives

$$\begin{aligned} P(\phi < \theta|D) = &\int_0^1 d\theta A_\theta \theta^{r_\theta}(1-\theta)^{n-r_\theta} \int_0^\theta d\phi (n+1)(1-\phi)^n \\ = &\int_0^1 d\theta A_\theta \theta^{r_\theta}(1-\theta)^{n-r_\theta}(1-(1-\theta)^{n+1}) \\ = &\, 1 - \int_0^1 d\theta A_\theta \theta^{r_\theta}(1-\theta)^{n-r_\theta}(1-\theta)^{n+1} \\ = &\, 1 - \int_0^1 d\theta A_\theta \theta^{r_\theta}(1-\theta)^{m-r_\theta}, \end{aligned} \tag{12}$$

where $m = 2n + 1$. Using the normalisation constants A_θ, this is

$$P(\phi < \theta|D) = 1 - \frac{(n+1)!}{(n-r_\theta)!}\frac{(m-r_\theta)!}{(m+1)!}. \tag{13}$$

For large n this can be approximated by

$$P(\phi < \theta|D) \approx 1 - \left(\frac{1}{2}\right)^{r_\theta+1} \tag{14}$$

For this work we can interpret $n = 10$ as the total number of TPS runs, and $r_\theta = 7$ as the number of observed switches in these 10 runs. Since n is not very large we have to use 13, which gives

$$P(\phi < \theta|\{7, 0, 10\}) = 0.99948 \tag{15}$$

Thus the odds that the hypotheses $\phi < \theta$ is correct, are about 2000 :1. So indeed, the outside channel is more likely than the inside channel.

References

1. Hoogsteen,K. (1959) The structure of crystals containing a hydrogen-bonded complex of 1-methylthymine and 9-methyladenine. *Acta Crystallogr*, **12**, 822–823.
2. Gould,I.R. and Kollman,P.A. (1994) Theoretical investigation of the hydrogen bond strengths in guanine-cytosine and adenine-thymine base pairs. *J. Am. Chem. Soc.*, **116**, 2493–2499.
3. Nikolova,E.N., Kim,E., Wise,A.A., O'Brien,P.J., Andricioaei,I., and Al-Hashimi,H.M. (2011) Transient Hoogsteen base pairs in canonical duplex DNA. *Nature*, **470**, 498–502.
4. Courtois,Y., Fromageot,P., and Guschlbauer,W. (1968) Protonated polynucleotide structures. 3. An optical rotatory dispersion study of the protonation of DNA. *Eur J Biochem*, **6**, 493–501.
5. Brovarets,O.O. and Hovorun,D.M. (2013) Can tautomerization of the A·T Watson–Crick base pair via double proton transfer provoke point mutations during DNA replication? A comprehensive QM and QTAIM analysis. *J. Biomol. Struct. Dyn.*, **32**, 127–154.
6. Brovarets',O.O., Yurenko,Y.P., and Hovorun,D.M. (2013) Intermolecular CH···O/N h-bonds in the biologically important pairs of natural nucleobases: A thorough quantum-chemical study. *J. Biomol. Struct. Dyn.*, **32**, 993–1022.
7. Yurenko,Y.P., Zhurakivsky,R.O., Samijlenko,S.P., and Hovorun,D.M. (2011) Intramolecular CH. . . O hydrogen bonds in the AI and BI DNA-like conformers of canonical nucleosides and their Watson–Crick pairs. quantum chemical and AIM analysis. *J. Biomol. Struct. Dyn.*, **29**, 51–65.
8. Macke,T.J. and Case,D.A. (1997) Modeling unusual nucleic acid structures. In Leontes,N. and SantaLucia,Jr.,J. (eds.), ACS Symposium Series. American Chemical Society, Washington, DC pp. 379–393.
9. Berman,H.M. (2000) The protein data bank. *Nucleic Acids Res.*, **28**, 235–242.
10. Duan,Y., Wu,C., Chowdhury,S., Lee,M.C., Xiong,G., Zhang,W., Yang,R., Cieplak,P., Luo,R., Lee,T., Caldwell,J., Wang,J., and Kollman,P. (2003) A point-charge force field for molecular mechanics simulations of proteins based on condensed-phase quantum mechanical calculations. *J. Comput. Chem.*, **24**, 1999–2012.
11. Jorgensen,W.L., Chandrasekhar,J., Madura,J.D., Impey,R.W., and Klein,M.L. (1983) Comparison of simple potential functions for simulating liquid water. *J. Chem. Phys.*, **79**, 926–935.
12. Darden,T., York,D., and Pedersen,L. (1993) Particle mesh Ewald: An Nlog(N) method for Ewald sums in large systems. *J. Chem. Phys.*, **98**, 10089–10092.
13. Essmann,U., Perera,L., Berkowitz,M.L., Darden,T., Lee,H., and Pedersen,L.G. (1995) A smooth particle mesh Ewald method. *J. Chem. Phys.*, **103**, 8577–8593.
14. Bussi,G., Donadio,D., and Parrinello,M. (2007) Canonical sampling through velocity rescaling. *J. Chem. Phys.*, **126**, 014101.
15. Parrinello,M. and Rahman,A. (1981) Polymorphic transitions in single crystals: A new molecular dynamics method. *J. Appl. Phys.*, **52**, 7182–7190.
16. Pronk,S., Páll,S., Schulz,R., Larsson,P., Bjelkmar,P., Apostolov,R., Shirts,M.R., Smith,J.C., Kasson,P.M., van der Spoel,D., Hess,B., and Lindahl,E. (2013) GROMACS 4.5: A high-throughput and highly parallel open source molecular simulation toolkit. *Bioinformatics*, **29**, 845–854.
17. Song,K., Campbell,A.J., Bergonzo,C., de los Santos,C., Grollman,A.P., and Simmerling,C. (2009) An improved reaction coordinate for nucleic acid base flipping studies. *J. Chem. Theory Comput.*, **5**, 3105–3113.
18. Laio,A. and Parrinello,M. (2002) Escaping free-energy minima. *Proc. Natl. Acad. Sci. USA*, **99**, 12562–12566.
19. Tribello,G.A., Bonomi,M., Branduardi,D., Camilloni,C., and Bussi,G. (2014) PLUMED 2: New feathers for an old bird. *Comput. Phys. Commun.*, **185**, 604–613.
20. Dellago,C., Bolhuis,P.G., Csajka,F.S., and Chandler,D. (1998) Transition path sampling and the calculation of rate constants. *J. Chem. Phys.*, **108**, 1964–1977.
21. Bolhuis,P.G., Chandler,D., Dellago,C., and Geissler,P.L. (2002) Transition Path Sampling: Throwing ropes over rough mountain passes, in the dark. *Annu. Rev. Phys. Chem.*, **53**, 291–318.
22. Juraszek,J. and Bolhuis,P.G. (2006) Sampling the multiple folding mechanisms of trp-cage in explicit solvent. *Proc. Natl. Acad. Sci. USA*, **103**, 15859–15864.
23. Ivani,I., Dans,P.D., Noy,A., Pérez,A., Faustino,I., Hospital,A., Walther,J., Andrio,P., Goñi,R., Balaceanu,A., Portella,G., Battistini,F., Gelpí,J.L., González,C., Vendruscolo,M., Laughton,C.A., Harris,S.A., Case,D.A., and Orozco,M. (2015) Parmbsc1: A refined force field for DNA simulations. *Nat. Methods*, **13**, 55–58.
24. van Erp,T.S., Moroni,D., and Bolhuis,P.G. (2003) A novel path sampling method for the calculation of rate constants. *J. Chem. Phys.*, **118**, 7762–7774.
25. Rogal,J. and Bolhuis,P.G. (2008) Multiple state transition path sampling. *J. Chem. Phys.*, **129**, 224107.
26. Swenson,D.W.H. and Bolhuis,P.G. (2014) A replica exchange transition interface sampling method with multiple interface sets for investigating networks of rare events. *J. Chem. Phys.*, **141**, 044101.
27. Kumar,S., Rosenberg,J.M., Bouzida,D., Swendsen,R.H., and Kollman,P.A. (1992) The weighted histogram analysis method for free-energy calculations on biomolecules. i. the method. *J. Comput. Chem.*, **13**, 1011–1021.
28. Swenson,D.W.H., Prinz,J.H., Noe,F., Chodera,J.D., and Bolhuis,P.G. (2019) OpenPathSampling: A Python framework for path sampling simulations. 1. Basics. *J. Chem. Theory Comput.*, **15**, 813–836.
29. van Erp,T.S. (2007) Reaction rate calculation by parallel path swapping. *Phys. Rev. Lett.*, **98**, 268301.
30. Pérez de Alba Ortíz,A., Vreede,J., and Ensing,B. (2019) The adaptive path collective variable: A versatile biasing approach

to compute the average transition path and free energy of molecular transitions. In Bonomi,M. and Camilloni,C. (eds.), Biomolecular Simulations: Methods and Protocols. Springer, New York, NY pp. 255–290.

31. Stelzl,L.S. and Hummer,G. (2017) Kinetics from replica exchange molecular dynamics simulations. *J. Chem. Theory Comput.*, **13**, 3927–3935.
